# Brain Tumor promotes axon growth across the midline through interactions with the microtubule stabilizing protein Apc2

**DOI:** 10.1371/journal.pgen.1007314

**Published:** 2018-04-04

**Authors:** Elise Arbeille, Greg J. Bashaw

**Affiliations:** Department of Neuroscience, Perelman School of Medicine, University of Pennsylvania, Philadelphia, PA, United States of America; New York University, UNITED STATES

## Abstract

Commissural axons must cross the midline to establish reciprocal connections between the two sides of the body. This process is highly conserved between invertebrates and vertebrates and depends on guidance cues and their receptors to instruct axon trajectories. The DCC family receptor Frazzled (Fra) signals chemoattraction and promotes midline crossing in response to its ligand Netrin. However, in Netrin or *fra* mutants, the loss of crossing is incomplete, suggesting the existence of additional pathways. Here, we identify Brain Tumor (Brat), a tripartite motif protein, as a new regulator of midline crossing in the *Drosophila* CNS. Genetic analysis indicates that Brat acts independently of the Netrin/Fra pathway. In addition, we show that through its B-Box domains, Brat acts cell autonomously to regulate the expression and localization of Adenomatous polyposis coli-2 (Apc2), a key component of the Wnt canonical signaling pathway, to promote axon growth across the midline. Genetic evidence indicates that the role of Brat and Apc2 to promote axon growth across the midline is independent of Wnt and Beta-catenin-mediated transcriptional regulation. Instead, we propose that Brat promotes midline crossing through directing the localization or stability of Apc2 at the plus ends of microtubules in navigating commissural axons. These findings define a new mechanism in the coordination of axon growth and guidance at the midline.

## Author summary

The establishment of neuronal connections that cross the midline of the animal is essential to generate neural circuits that coordinate the left and right sides of the body. Axons that cross the midline to form these connections are called commissural axons and the molecules and mechanisms that control midline axon crossing are remarkably conserved across animal evolution. In this study we have used a genetic screen in the fruit fly in an attempt to uncover additional players in this key developmental process, and have identified a novel role for the Brain Tumor (Brat) protein in promoting commissural axon growth across the midline. Unlike its previous described functions, in the context of midline axon guidance Brat cooperates with the microtubule stabilizing protein Apc2 to coordinate axon growth and guidance. Molecular and genetic analyses point to the conserved B box motifs of the Brat protein as key in promoting the association of Apc2 with the plus ends of microtubules. Brat is highly conserved and future studies will determine whether homologous genes play analogous roles in mammalian neural development.

## Introduction

Organisms with bilateral symmetry coordinate the left and right sides of their body by establishing reciprocal connections in the central nervous system. During development, commissural axons navigate across the midline to form contralateral connections by responding to attractant and repellant cues expressed at the midline and in other cells [1]. To alter growth cone motility, guidance receptors must signal to the underlying growth cone cytoskeleton [2]. Midline and ventricular zone-derived Netrin and its receptor DCC (Deleted in Colorectal Carcinoma) or Fra (Frazzled) in *Drosophila*, are a highly conserved chemoattractive guidance pathway [3–5]. Loss of DCC leads to profound commissural axon guidance defects, as well as developmental and movement disorders [6–10]. Despite the clear importance of Netrin signaling in promoting axon growth across the midline, many axons still cross the midline in *netrinAB* double mutants or *fra* mutants in *Drosophila*. This indicates that additional pathways must promote midline crossing [11,12]. Indeed, several additional factors implicated in promoting midline axon attraction have been identified in invertebrate and vertebrate systems, such as Shh/Boc [13], Sema2a/Sema1a [14], and VEGF/Flk1 [15]. In addition, many other mechanisms have been described that promote axon growth across the midline by preventing premature responses to repulsive molecules secreted from the midline, such as Slits and Semaphorins [16–20]. Despite this progress, it is clear from genetic analysis that additional pathways are likely to be required to ensure the precise regulation of midline circuit formation.

To identify additional pathways implicated in the midline crossing process, we performed a genetic screen using a sensitized genetic background. In this background a dominant negative Fra receptor (FraΔC- missing its entire cytoplasmic domain) is expressed in a subset of commissural neurons in the *Drosophila* embryo resulting in an easily quantifiable defect in midline crossing [21]. From this screen, we identified Brain Tumor (Brat), as a new regulator of midline crossing. Brat belongs to the tripartite motif (TRIM)-NHL family of proteins and is conserved throughout evolution from *C*. *elegans* to humans. Brat contains two B-box domains (BB), a Coiled-coil domain (CC) in the N-terminus, and a NHL domain in the C-terminus [22]. Identified first as a translational repressor [23], Brat has been shown to play important roles in various biological processes, such as in the regulation of microRNA activity during development [24,25], and in the control of the proliferation and differentiation of specific neural precursor lineages during early neural development [26]. Moreover, previous studies showed that each domain executes distinct and specific functions. The NHL domain is essential to suppress the translation of *hunchback* mRNA in the posterior part of the embryo during early development [27], and for the maintenance of mushroom body axon connections [28]. In addition, it has recently been shown that during neurogenesis Brat regulates asymmetric protein segregation through the CC domain and specifies intermediate neural progenitor (INP) identity via its B-box domains [29]. This process involves Apc2, a key component of the destruction complex in the canonical Wnt signaling pathway [30]. The destruction complex attenuates the transcriptional activity of armadillo/βcatenin to prevent the activation of Wnt target genes, and thereby promotes the self-renewal of intermediate progenitors.

Interestingly, in addition to its role in the destruction complex, Apc2 is a microtubule plus-end binding protein (+TIP) [31,32]. In *Drosophila* sensory neuron dendrites, Apc2 interacts with EB1 (for End Binding) to control microtubule polarity [31]. In growth cones, APC, the vertebrate homologue of *Drosophila* Apc2 [33], regulates axonal projections and changes in axon behavior by regulating microtubule stability and growth directionality [34,35]. In this context, tethered to the microtubule plus-ends, APC allows active axon elongation by linking microtubules to the leading edge of the growth cone.

In this study, we report that Brain Tumor maintains Apc2 at the plus-ends of microtubules to promote axon elongation and midline crossing. Brat acts independently of the Fra/Netrin pathway and independently of its common partners Pumilio, Nanos and d4EHP, which are required for the inhibition of mRNA translation. In addition, we show that this process requires the B-Box domains of Brain Tumor. Reducing the function of Apc2 in *brat* mutants, results in enhanced commissural guidance defects in the FraΔC sensitized background. Moreover, Apc2 expression and localization are altered in *brat* mutant embryos suggesting that Brat function in this context is critically dependent on Apc2. These data suggest a model where Brat promotes the elongation of the axon before crossing by maintaining Apc2 at the microtubule plus-ends.

## Results

### A genetic screen identifies a role for Brain Tumor in promoting midline crossing

**Fig 1:**
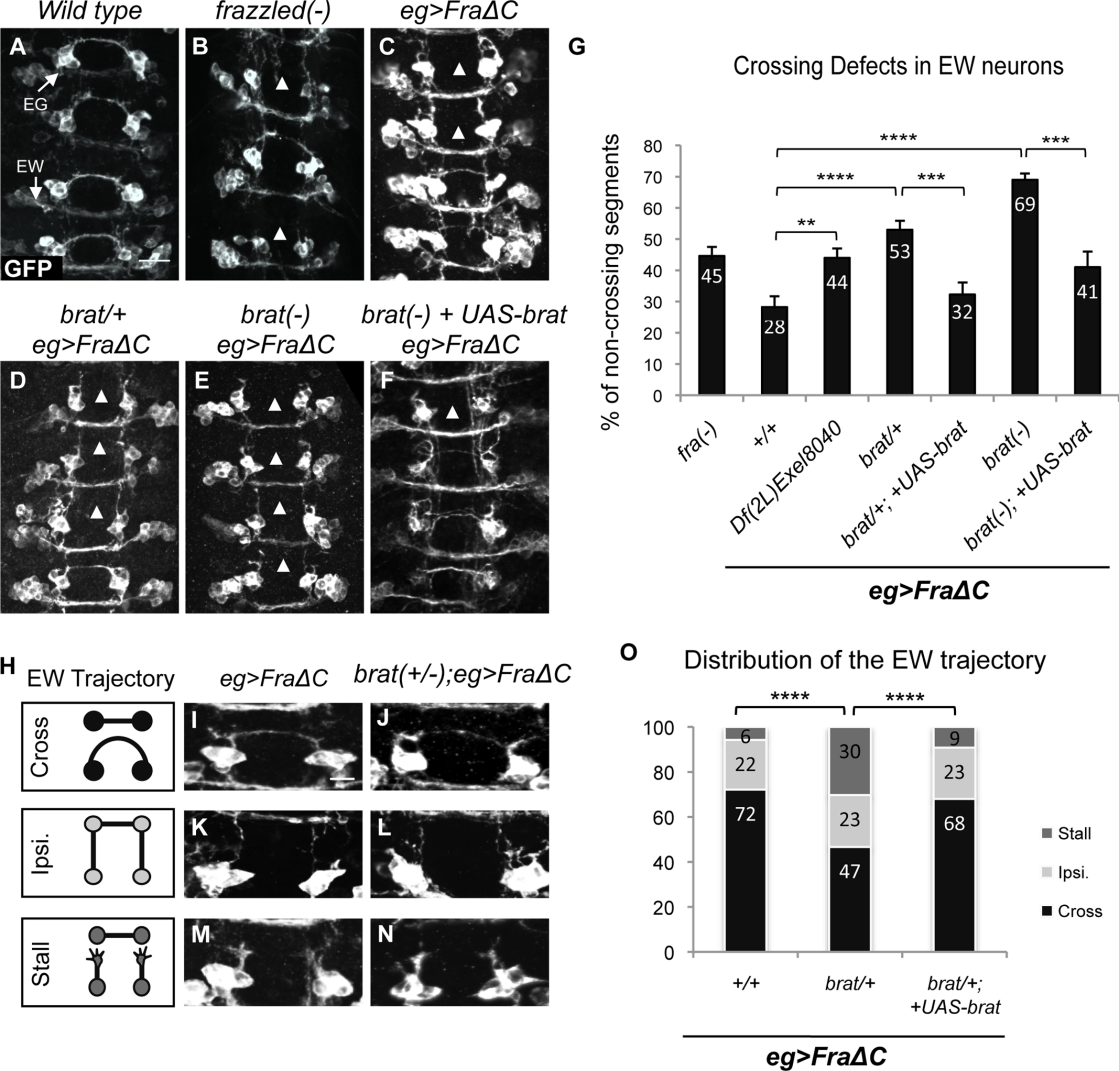
Brain Tumor is a positive regulator of midline crossing. (A-F) Stage 15–16 embryos of the indicated genotypes carrying eg-GAL4 and UAS-tauMycGFP transgenes, stained with anti-GFP antibody. Anti-GFP reveals cell bodies and axons of the eagle neurons (EG and EW). Anterior is up in all images. Scale bar represents 10μm (A). EG neurons project through the anterior commissure of each segment, while EW neurons project through the posterior commissure. Arrowheads indicate segments with non-crossing EW axons. (A) In wild-type embryos EW axons cross in the posterior commissure in 100% of segments. (B) In *fra* mutants EW axons fail to cross in 36% of segments (arrowheads). (C) EW axons fail to cross in 28% of segments when UAS-FraΔC is selectively expressed in eagle neurons. (D) In a FraΔC background the heterozygosity for *brat* enhances the EW crossing defects to 53%. (E) Complete loss of *brat* enhances the EW crossing defect to 69% of segments in FraΔC background. (F) EW crossing defects in *brat/brat;* FraΔC embryos are rescued (69% versus 41%) when UAS-Brat is expressed in eagle neurons. (G) Quantification of EW midline crossing defects in the genotypes shown in (B-F). Df (2L) Exel8040 is a chromosomal deficiency containing *brat*. Data are presented as mean ± SEM. 20 embryos were scored for each genotype. Significance was assessed by multiple comparisons using ANOVA (^∗∗∗^p < 0.001). (H) Schematic diagrams of the EW axon trajectories observed in each genotype; the EW axons can cross the midline (Cross), grow ipsilaterally (Ipsi) or stall (Stall). (I, K, M) When UAS-FraΔC is selectively expressed in eagle neurons, 72% of the EW axons cross the midline (I), 22% grow ipsilaterally (K) and 6% stall (M). (J,L,N) Heterozygosity for *brat* in a FraΔC background enhances the EW crossing defects, 47% of the EW cross the midline (J), 23% grow ipsilaterally (L) and 30% stall (N). (O) Quantification of the distribution of the EW axon trajectories in the genotypes shown in (I-N). The enhanced EW crossing defects in *brat/+;* FraΔC embryos are rescued when UAS-Brat is expressed in eagle neurons. Data are presented as mean ± SEM. 20 embryos were scored for each genotype. Significance was assessed using Chi-squared test (****p < 0.0001).

In order to identify new molecules and factors implicated in midline crossing, we performed a genetic modifier screen using a truncated Fra receptor (FraΔC) missing its cytoplasmic domain that functions as a dominant negative [11]. By targeting the expression of the FraΔC transgene to a small subset of commissural neurons, the eagle neurons, we are able to generate a highly sensitized background. The eagle neuron population is comprised of two pools of neurons, the EWs and the EGs, which are found in each hemisegment. Around ten EG neurons project their axons through the anterior commissure, while only three EW neuron axons project through the posterior commissure [36] (Fig 1A and 1G). In *fra* mutants, EW axons fail to cross the midline in 36% of embryonic segments, while the axons of EG neurons are unaffected [11] (Fig 1B and 1G). A similar phenotype is observed in stage 16 *wild type* embryos expressing FraΔC specifically in eagle neurons (Fig 1C and 1G).

We started by screening large deficiencies covering a majority of the second chromosome and identified dominant enhancers of the FraΔC crossing defects (S1 Fig). One deficiency, Df(2L)Exel8040, significantly enhances the FraΔC phenotype resulting in 44% crossing defects (Fig 1G). After testing the different candidate genes present in this interval, we identified the enhancer as Brain Tumor (Brat). A null allele, *brat*^*11*^, fully recapitulates the enhanced EW defects observed in the deficiency. (Fig 1D and 1G). Moreover, when both copies of *brat* are removed in the FraΔC screening background, EW crossing defects are strongly enhanced to 69% (Fig 1E and 1G). Importantly, this mutant phenotype can be rescued when full-length Brat (UAS-Brat) is expressed selectively in eagle neurons (Fig 1F and 1G), suggesting that Brat functions in commissural axons to promote midline crossing. To determine whether the crossing defects are a consequence of a failure of axon growth or a failure to turn toward the midline, we carefully examined the trajectory of EW axons in these embryos and observed several qualitatively distinct phenotypes (Fig 1H). In addition to crossing the midline normally, EW axons can either continue to grow ipsilaterally and fail to turn, or they can stall before or during midline crossing. For example, when UAS-FraΔC is expressed, EW axons cross in 72% of embryonic segments (Fig 1O and 1I), continue to grow ipsilaterally in 22% of segments (Fig 1O and 1K) and stall in only 6% of segments (Fig 1O and 1M). In the FraΔC background, heterozygosity for *brat* enhances the EW crossing defects, decreasing the proportion EW crossing axons to 47% (Fig 1O and 1J) and increasing the proportion of stalled axons to 30% (Fig 1O and 1N). However, the proportion of the ipsilaterally growing axons remains the same (22%) (Fig 1O and 1L), suggesting that the increase of the crossing defects is due to more stalled axons when one copy of *brat* is removed. The distribution of the EW axon trajectories can be rescued when full-length Brat (UAS-Brat) is expressed selectively in eagle neurons (Fig 1O), restoring the proportion observed in the FraΔC background. These results strongly suggest a cell autonomous role for *brat* in promoting midline crossing and axon growth.

### Brat promotes midline crossing independently of the Fra-Netrin pathway

**Fig 2:**
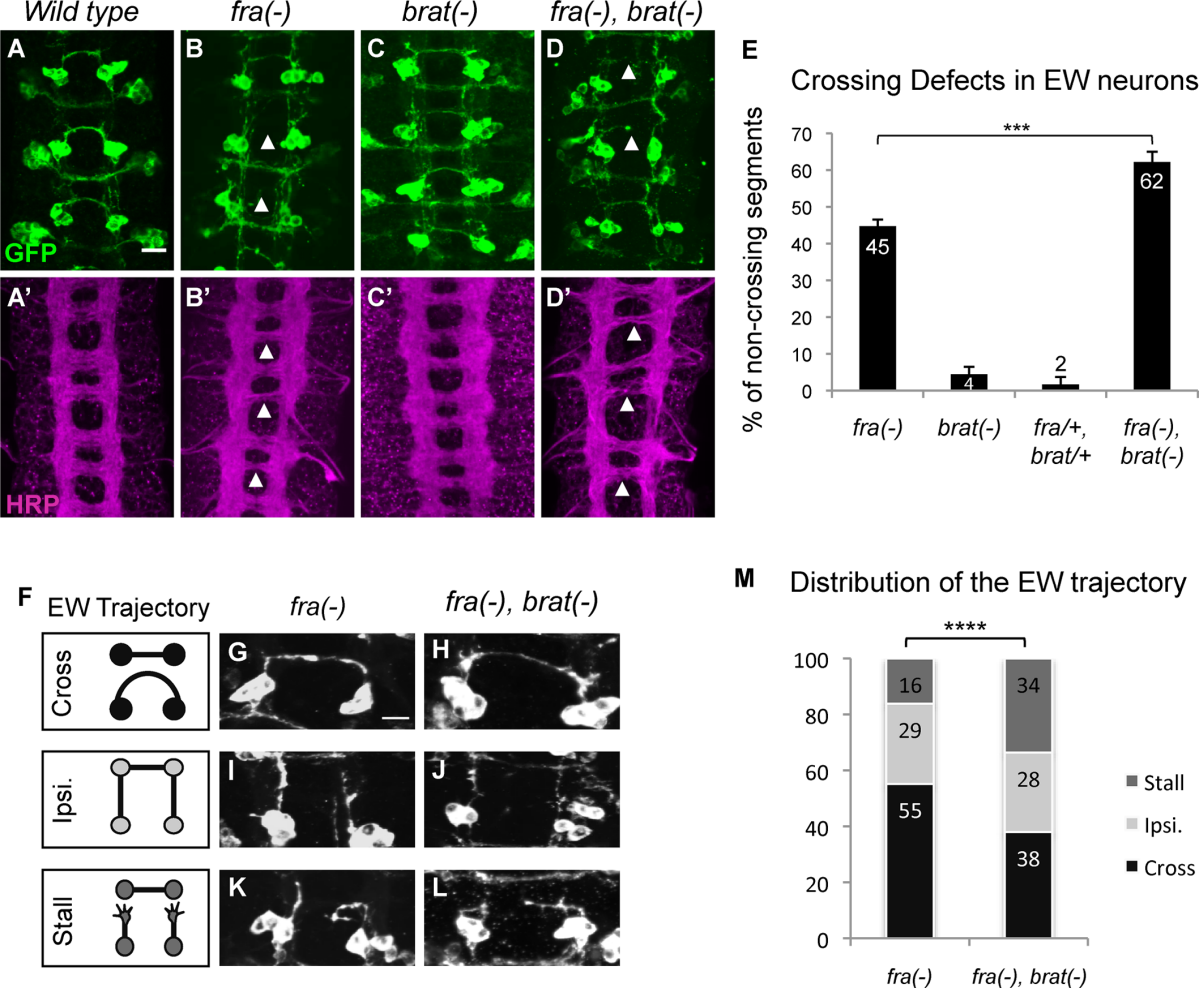
Brat acts in parallel to the *Netrin-Fra* pathway. (A-D) Stage 15–16 embryos of the indicated genotypes carrying eg-GAL4 and UAS-tauMycGFP transgenes, stained with anti-GFP (green) (A-D) or anti-HRP (magenta) (A’-D’) antibodies. Anti-GFP labels cell bodies and axons of the eagle neurons (EG and EW), Anti-HRP reveals all of the CNS axons. Scale bar represents 10μm (A). Arrowheads indicate segments with non-crossing EW axons (A-D) or thin commissures (A’-D’). (A) EW neurons cross in the posterior commissure in 100% of segments in wild-type embryos. (A’) In every segment thick anterior and posterior commissures are formed as axons cross the midline. (B) In *fra* mutants EW neurons fail to cross in 36% of segments. (B’) *fra* mutants show thinner commissures. (C) and (C’) *brat* homozygous mutants show no obvious signs of commissural guidance defects: EW neurons fail to cross in only 4% of segments. (D) In *fra*, *brat* double mutants EW axons fail to cross the midline in 56% of segments. (D’) *fra*, *brat* double mutants also show thinner commissures. (E) Quantification of EW midline crossing defects in the genotypes shown in (B-D). Data are presented as mean ± SEM. 20 embryos were scored for each genotype. Significance was assessed by multiple comparisons using ANOVA (^∗∗∗∗^p < 0.0001). (F) Schematic diagrams of the EW axon trajectories observed in each genotype; the EW axons can cross the midline (Cross), grow ipsilaterally (Ipsi) or stall (Stall). (G, I, K) In *fra* mutants, 55% of the EW axons cross the midline (G), 29% grow ipsilaterally (I) and 16% remain stalled (K). (H, J, L) In *fra*, *brat* double mutants, 38% of the EW axons cross the midline (H), 28% grow ipsilaterally (J) and 34% stall (L). (M) Quantification of the distribution of the EW axon trajectories in the genotypes shown in (G-L). Data are presented as mean ± SEM. 20 embryos were scored for each genotype. Significance was assessed using Chi-squared test (****p < 0.0001).

Previously, we showed that Brat enhances the EW axon crossing defects induced by FraΔC. Since *brat* mRNA is expressed and functions in commissural neurons during axon growth and midline crossing, it is a good candidate to interact with Fra in this process (Fig 1 and S1 Fig). To test if Brat functions together with or independently of the Netrin-Fra pathway, we examined genetic interactions between *brat* and *fra* mutants. We scored EW axon crossing defects, as well as the pattern of the entire axon scaffold stained with anti-horse radish peroxidase (HRP) antibody, in *fra* mutants, *brat* mutants and *brat*, *fra* double mutants. In *wild type* embryos, HRP staining reveals that thick anterior and posterior commissures form in each segment and GFP staining reveals that EW and EG axons cross the midline (Fig 2A and 2E), (Fig 2A’). In *fra* mutants, the EW neurons fail to extend axons across the midline in 45% of segments (Fig 2B and 2E) and a significant crossing defect is also observed when all CNS axons are visualized (Fig 2B’). In contrast, *brat* zygotic null mutants show no significant crossing defects in either eagle neurons or in the axon scaffold (Fig 2C, 2C’ and 2E), suggesting that *brat* is likely to act redundantly to promote crossing. If *brat* and *fra* are functioning in the same pathway, we would expect to find the same extent of crossing defects in *fra* mutants and *brat*, *fra* double mutants. In contrast, enhancement of the defects observed in *fra* mutants would be expected in the *brat*, *fra* double mutants if *brat* and *fra* function in independent pathways. While trans-heterozygous embryos for *brat* and *fra* display no defects (Fig 2E), the double mutants enhance the EW crossing defect to 62% and thinner commissures are observed in the axon scaffold (Fig 2D, 2D’ and 2E). When we carefully analyze the trajectory of the EW axons in these genotypes, we observe the same categories of phenotypes described above: axons can cross the midline, continue to grow ipsilaterally and fail to turn, or stall before or during midline crossing (Fig 2F). In *fra* mutants, 55% of the EW axons cross the midline (Fig 2G and 2M) while in *brat*, *fra* double mutants, this proportion is reduced to 38% (Fig 2H and 2M). The proportion of axons that grow ipsilaterally remains the same in the both genotypes with 28% in the *fra* mutants and 29% in the *brat*, *fra* double mutants (Fig 2I, 2J and 2M). However, the proportion of stalled axons observed increases from 16% to 34% in the double mutants (Fig 2K, 2L and 2M), suggesting that the increase of crossing defects is due to more stalled axons in the absence of Brat. Moreover, while the overexpression of *brat* in all neurons does not induce ectopic midline crossing (S2A–S2B’ Fig), *brat* expression in the eagle neurons can significantly suppress the non-crossing phenotype observed in the FraΔC background (S2C–S2E Fig). These phenotypes strongly support a role for Brat in axon guidance and indicate that Brat must function independently of the Netrin-Fra pathway to promote midline crossing and axon elongation.

### Brat controls axon guidance independently of the Nanos/Pumilio complex and d4EHP

**Fig 3:**
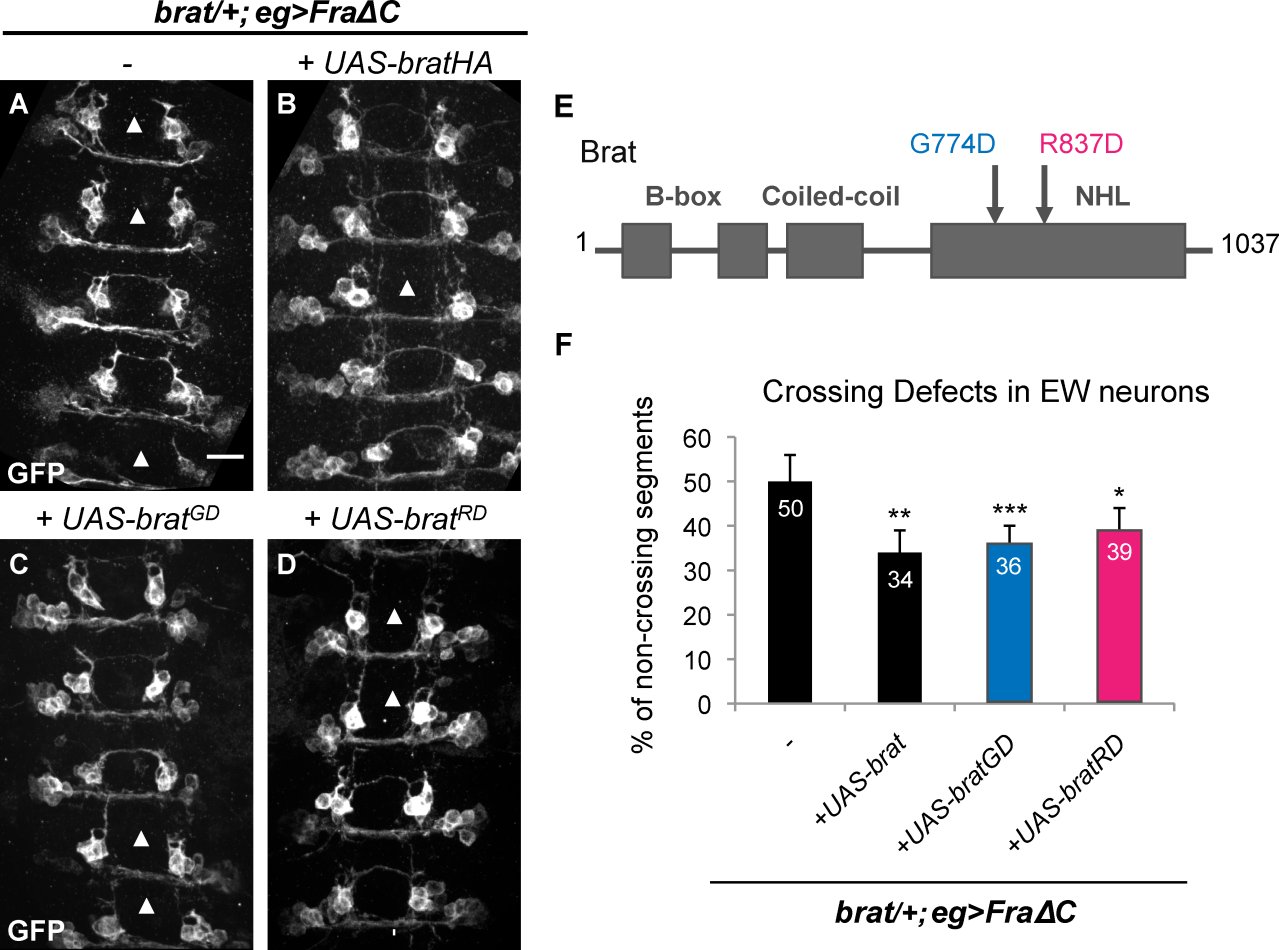
Brat acts independently of the Nanos/Pumilio complex and of d4EHP. (A-D) Stage 15–16 embryos of the indicated genotype carrying eg-GAL4 and UAS-tauMycGFP transgenes, stained with anti-GFP antibody. Anti-GFP labels cell bodies and axons of the eagle neurons (EG and EW). Scale bar represents 10μm (A). Arrowheads indicate segments with non-crossing EW axons. (A) Heterozygosity for *brat* enhances the EW crossing defects to 50% in a FraΔC background. (B-D) EW crossing defects in heterozygous *brat* mutants expressing FraΔC are rescued when (B) UAS-Brat (50% versus 34%), (C) UAS-Brat^GD^ (50% versus 36%) or (D) UAS-Brat^RD^ (50% versus 39%) are expressed in eagle neurons. (E) Schematic representation of Brat protein and its different domains. The G774D and R837D point mutations are indicated with arrows. (F) Quantification of EW midline crossing defects in the genotypes shown in (A-D). Data are presented as mean ± SEM. 20 embryos were scored for each genotype. Significance was assessed by multiple comparisons using ANOVA (^∗∗∗^p < 0.001).

In early embryonic development and in the larval peripheral nervous system, Brat cooperates with its cofactors Nanos (Nos) and Pumilio (Pum) to repress the translation of target mRNAs [23,37,38]. Thus, we next sought to determine whether Brat function during commissural axon guidance depends on the ability of Brat to interact with Nos and Pum. To address this question, we took advantage of previous studies that identified specific amino acid residues within the Brat NHL domain that are required for the association of Brat with Nos and Pum (Fig 3E) [23,39]. Interestingly, expression of *UASBrat*^*G774D*^ is just as efficient as wild-type *UASBrat* in restoring midline crossing in the *Fra*Δ*C*, *brat/+* sensitized background, suggesting that Brat functions independently of these factors during midline guidance (Fig 3A–3C and 3F). In addition, it has been shown that Brat can repress the translation of *hunchback* by interacting with d4EHP an EIF4e-related cap binding protein [37,40]. Expression of *UASBrat*^*R837D*^, which is unable to associate with d4EHP, also rescues the midline guidance defects (Fig 3D and 3F). Importantly, we verified that the relative levels and localization of the HA-tagged Brat transgenes used in these rescue experiments are equivalent by visualizing transgene expression in Eg neurons using anti-HA immunostaining (S3A–S3C Fig).

**Fig 4:**
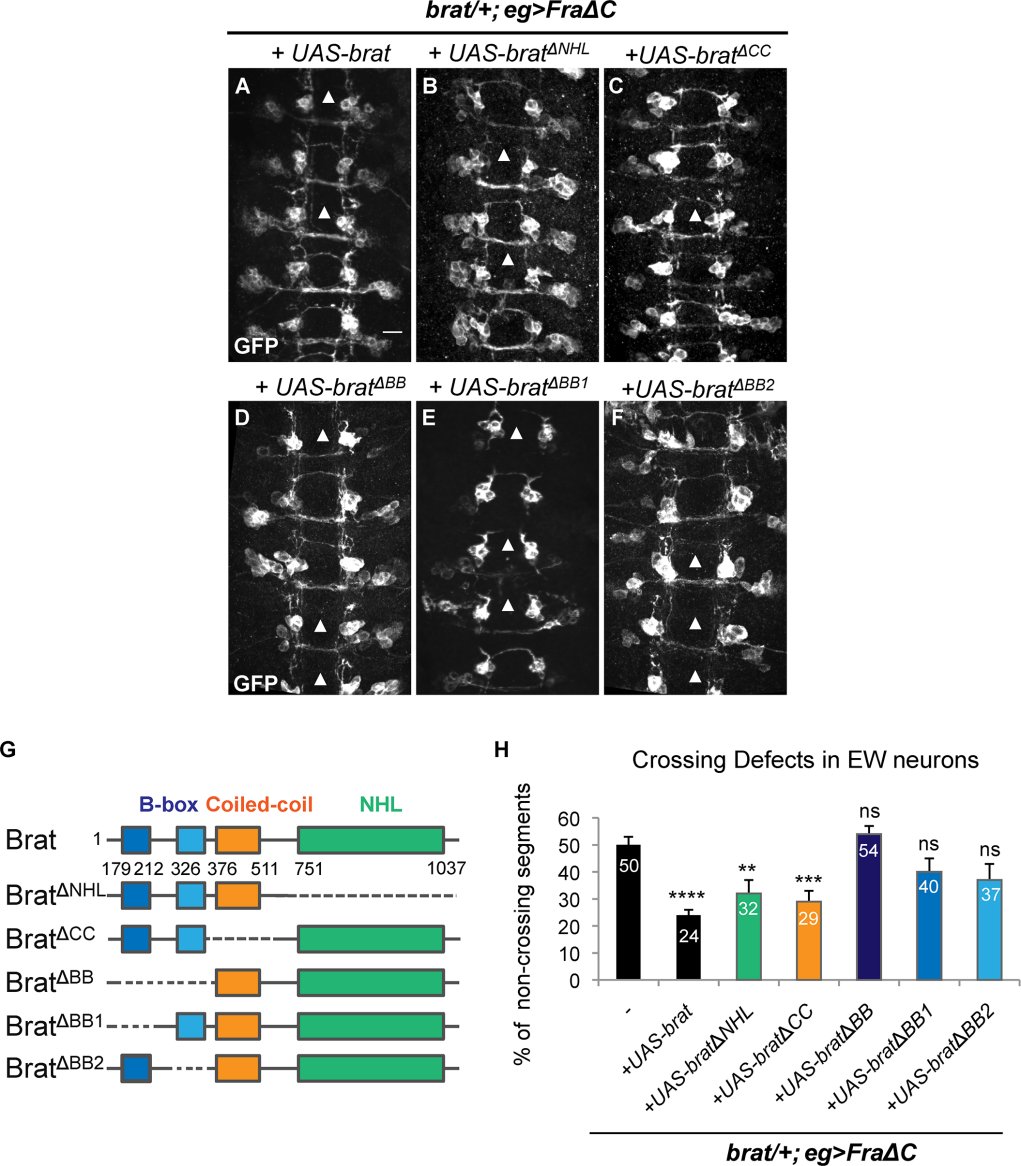
The B-box domains of Brat are required for its midline crossing function. (A-F) Stage 15–16 embryos of the indicated genotype carrying eg-GAL4 and UAS-tauMycGFP transgenes, stained with anti-GFP antibody. Anti-GFP labels cell bodies and axons of the eagle neurons (EG and EW). Scale bar represents 10μm (A). Arrowheads indicate segments with non-crossing EW axons. (A-C) EW crossing defects in the heterozygous *brat* mutant expressing FraΔC are rescued when (A) UAS-Brat (50% versus 24%), (B) UAS-Brat^ΔNHL^ (50% versus 32%) or (C) UAS-Brat^ΔCC^ (50% versus 29%) are expressed in eagle neurons. (D-F) In the heterozygous *brat* mutant expressing FraΔC, expression of (D) UAS-Brat^ΔBB^, (E) UAS-Brat^ΔBB1^ or (F) UAS-Brat^ΔBB2^ fail to rescue the EW midline crossing defects (respectively for (D) (E) and (F): 50% versus 54%, 50% versus 40% and 50% versus 37%). (G) Schematic representation of Brat full-length protein and Brat deletion domain mutants used to identify the domain required for midline crossing. (H) Quantification of EW midline crossing defects in the genotypes shown in (A-F). Data are presented as mean ± SEM. 20 embryos were scored for each genotype. Significance was assessed by multiple comparisons using ANOVA (^∗∗∗∗^p < 0.0001).

These results would seem to preclude a role for Brat acting as a component of a translational repressor complex in the context of midline guidance; however, there is previously published evidence that Brat can regulate translation independently of the Nos/Pum complex [28]. Specifically, Brat has been shown to regulate the maintenance of mushroom body axons in the Drosophila brain, and this function appears to depend on the ability of Brat to attenuate the translation of the Src64B protein [28]. Similarly to what we have shown here for commissural axon guidance (Fig 3), the *UASBrat*^*G774D*^ or *UASBrat*^*R837D*^ variants can also fully rescue the mushroom body axon maintenance defects observed in *brat* mutants [28]. In addition, previous work from our lab indicates that Src64B acts to negatively regulate midline crossing, and that it does so independently of the Netrin-Fra pathway [41], raising the possibility that Brat may promote midline crossing by attenuating Src expression. Therefore, to directly investigate a link between Brat and Src64B during commissural axon guidance, we took advantage of a GFP reporter line that indicates the level of Src64B expression. In contrast to the elevated reporter expression observed in mushroom body axons in *brat* mutants, we observe no difference in Src64B levels in CNS axons in *brat* mutant embryos (S4 Fig). Finally, and in direct contrast to its role in mushroom body axon maintenance, we find that the NHL domain of Brat is dispensable for its midline axon guidance function, since UASBratΔ^NHL^ can rescue the EW crossing defects in the *Fra*Δ*C*, *brat/+* sensitized background just as well as wild-type (Fig 4B and 4H). Taken together these observations indicate that the mechanism underlying Brat activity in embryonic commissural axons is independent of translational regulation and distinct from its function in mushroom body axon maintenance.

### The B-box domains of Brat are required for its midline crossing function

In order to gain insight into the mechanism underlying Brat activity in commissural axon guidance, we carried out a series of structure function experiments to define the sequence requirements for Brat activity during commissural axon guidance. The results described above eliminated a possible role for the C-terminal NHL domain in this process, so we turned our attention to the N-terminal coiled-coil domain (CC), which is known to play a role in regulating asymmetric protein segregation, and the pair of B-box domains (BB1 and BB2), which have been implicated in the control of intermediate neural progenitor (INP) cell identity [29]. We used a series of previously described Myc-tagged UAS Brat transgenes bearing deletions in these domains and tested them in our midline crossing rescue assay (Fig 4G). In contrast to wild-type UAS Brat, UAS BratΔ^NHL^ and UAS BratΔ^CC^, all of which recued the EW crossing defects in the *Fra*Δ*C*, *brat/+* sensitized background (Fig 4A–4C and 4H), deletion of the B-box domains, either singly or in combination show no significant rescuing activity (Fig 4D–4F and 4H), although we did detect a trend indicating that the combined deletion of both B-boxes may cause a greater impairment in Brat function than single BB deletions. Importantly, we verified that the relative levels and localization of the Myc-tagged Brat transgenes used in these rescue experiments are equivalent by visualizing transgene expression in Eg neurons using anti-Myc immunostaining (S3D–S3I Fig).

### Midline crossing is sensitive to reduced Apc2 function

**Fig 5:**
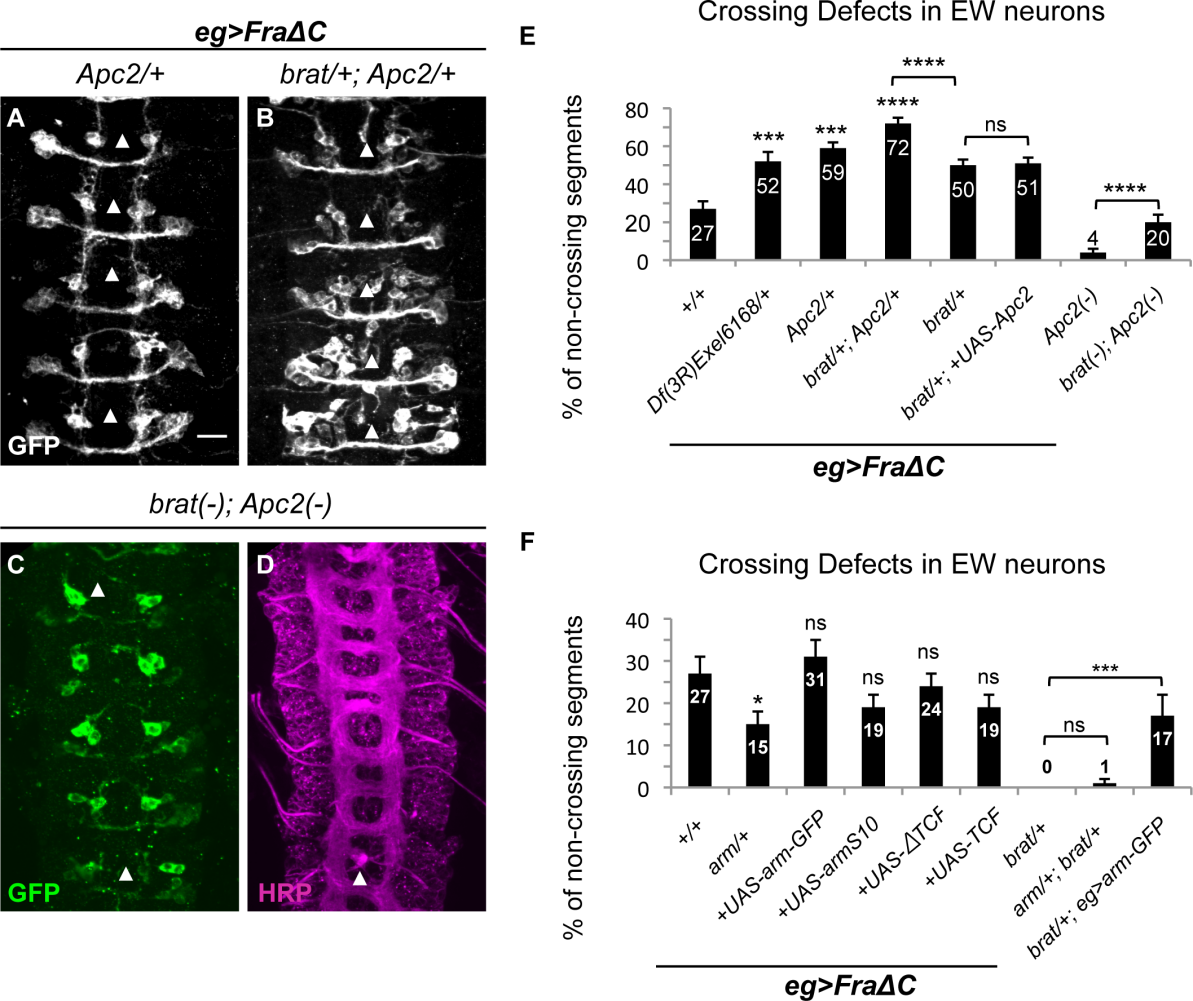
Midline crossing is sensitive to reduced Apc2 and Arm function and does not require Arm transcriptional activity. (A-D) Stage 15–16 embryos of the indicated genotype carrying eg-GAL4 and UAS-tauMycGFP transgenes, stained with anti-GFP (grey or green) (A-C) or anti-HRP (magenta) (D) antibodies. Anti-GFP labels cell bodies and axons of the eagle neurons (EG and EW), Anti-HRP reveals all of the CNS axons. Scale bar represents 10μm (A). Arrowheads indicate segments with non-crossing EW axons (A-C) or thin commissures (D). (A) In a FraΔC background the heterozygosity for *Apc2* enhances the EW crossing defects to 59%. (B) In the embryos double heterozygous for *Apc2* and *brat* expressing UAS-FraΔC selectively in eagle neurons, EW axons fail to cross in the posterior commissure in 72% of segments. (C) In *Apc2* and *brat* double mutant embryos, EW axons fail to cross in the posterior commissure in 20% of segments and show thinner commissures in some segments (D). (E) Quantification of EW midline crossing defects in the genotypes shown in (A-D). Df (2L) Exel6168 is a chromosomal deficiency containing *Apc2*. Data are presented as mean ± SEM. 20 embryos were scored for each genotype. Significance was assessed by multiple comparisons using ANOVA (^∗∗∗∗^p< 0.0001). (F) Quantification of EW midline crossing defects in the indicated genotypes. Data are presented as mean ± SEM. 20 embryos were scored for each genotype. Significance was assessed by multiple comparisons using ANOVA (^∗∗∗^p < 0.001).

Given the observation that the role of Brat in regulation of INP identity depends on its B-box domains and that in this context Brat interacts with components of the Wnt signaling pathway, we next investigated whether its role in commissural axon guidance could share common mechanistic features. Consistent with findings in the study of the specification of INP identity, reducing the function of *Apc2*, a component of the destruction complex, either with specific point mutations or with a deletion of the *Apc2* locus, significantly enhances the EW crossing defects in the *Fra*Δ*C* sensitized background (Fig 5A and 5E), while reducing the function of the Drosophila B-catenin Armadillo (Arm) leads to a reciprocal effect and significantly suppresses EW crossing defects caused by the FraΔC transgene (Fig 5F). Furthermore, reducing the function of *Apc2* in eagle neurons in *brat* mutants expressing FraΔC further enhances the commissural guidance defects to 72% (Fig 5B and 5E). We also tested whether expressing UAS-Apc2 selectively in eagle neurons in the FraΔC background could suppress the effect of reducing *brat*, but this manipulation had no effect on the midline crossing phenotype (Fig 5E). Together, these observations are consistent with the hypothesis that Brat promotes midline crossing by cooperating with Apc2 to limit the activity of Arm, potentially by attenuating Arm-activated gene transcription. This hypothesis makes a number of explicit predictions about the consequences of manipulating components of the Wnt pathway on EW axon midline crossing and we therefore conducted a series of genetic interaction experiments to test this model.

First, if Apc2 is acting to attenuate Arm activity, we predicted that over-expressing Apc2 should lead to a similar outcome to decreasing Arm function, resulting in a suppression of the FraΔC phenotype. However, we find that over-expressing Apc2 does not have any effect on the EW crossing phenotype in the FraΔC background (Fig 5E). Similarly, we expected that over-expressing UAS-Arm or a mutant variant of Arm that leads to elevated protein stability, UAS-ArmS10 [42], should lead to a significant enhancement of the EW midline crossing defects; however, these manipulations have no effect on the midline crossing phenotypes (Fig 5F). We also targeted downstream components of the Arm pathway that are specifically required for Arm-dependent gene transcription, reasoning that if the strong suppression of the midline crossing defects observed in *arm* loss of function results from a failure of Arm-dependent gene expression, then blocking transcriptional activity with dominant negative variants of TCF should also suppress the midline crossing defects. However, as we found for Arm and Apc2 over-expression, expressing multiple different TCF transgenes or TCF dominant negative transgenes had no impact on the EW crossing defects (Fig 5F). The same set of genetic manipulations using many of the same transgenic lines gave very different outcomes in the context of Brat-mediated regulation of INP identity, where results are consistent with the model that the primary role of Brat in INPs is to antagonize Armadillo activity [29]. In the context of axon elongation and guidance, it is clear that the experiments described here do not support the idea that Brat and Apc2 regulate midline guidance through antagonizing Arm-dependent gene expression.

Despite the fact that the results described above argue strongly against the idea that Brat and Apc2 regulate axon growth and guidance through antagonizing Arm-dependent gene expression, the fact that reducing *arm* function suppresses the midline crossing defects in the FraΔC background point to a possible contribution of Arm to midline axon guidance. Therefore, to further explore a relationship between Arm and Brat, we analyzed the effect of modulating Arm levels in embryos that are heterozygous for mutations in *brat*. Interestingly, while *brat*, *arm* compound heterozygotes have no crossing defects, there is a significant reduction in midline crossing when UAS-Arm is expressed in *brat* heterozygotes (Fig 5F). These observations could suggest that Arm may act to inhibit Brat function; however, given that the complete removal of zygotic *brat* does not result in any guidance defects, this explanation does not seem likely. We favor the alternative possibility that Arm may impinge on a parallel pathway to prevent crossing. Here, it is intriguing to note that the similar over-expression of Arm in the FraΔC genetic background does not enhance the crossing defects, suggesting a possible role for Arm in modulating Fra-dependent axon attraction. It will be interesting in the future to explore potential genetic and biochemical links between Arm and Fra.

### Brat and Apc2 cooperate to promote midline axon guidance

The observation that simultaneous heterozygosity for both *Apc2* and *brat* leads to significantly stronger midline crossing defects in the FraΔC background than heterozygosity for either *brat* or *Apc2* alone is consistent with the idea that they work together to promote midline crossing. We also examined the consequences of removing both copies of *Apc2* in an otherwise wild-type background. Similar to our findings with *brat* zygotic null mutants, *Apc2* zygotic null mutants show no significant crossing defects in either eagle neurons or in the axon scaffold (Figs 2C, 2C’, 2E and 5E). Since both Apc2 and Brat are maternally deposited, we reasoned that the simultaneous removal of the zygotic copies of both of these genes might sufficiently limit Brat and Apc2 function to reveal defects in midline crossing in an otherwise wild-type background. Indeed, the double mutants for *brat* and *Apc2* show significant crossing defects in EW axons (Fig 5C, 5D and 5E). In addition, *brat*, *Apc2* double mutants also lead to additional disruptions to the axon scaffold that could reflect roles in processes other than midline crossing. Together with the dose-dependent genetic interactions, these observations further support an important role for Brat and Apc2 in promoting axon growth across the midline.

### Apc2 expression in EW neurons is reduced in *brat* mutant embryos

**Fig 6:**
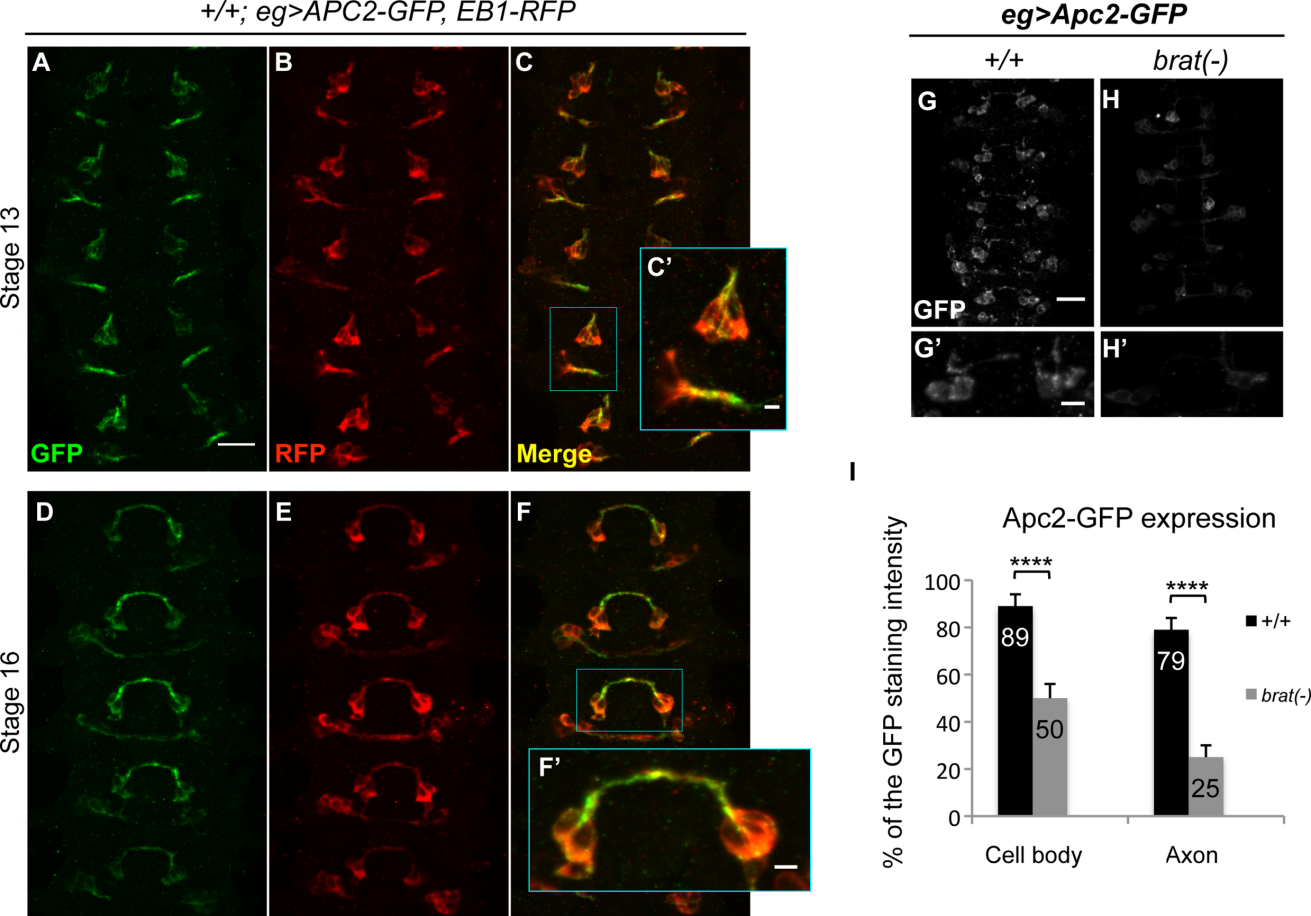
Apc2 expression co-localizes with EB1 in growing axon and cell bodies of Eagle neurons and is reduced in *brat* mutant embryos. (A-F’) Stage 13 and 16 embryos carrying eg-GAL4, UAS-Apc2GFP and UAS-EB1RFP transgenes, stained with anti-GFP (green) and anti-RFP (red) antibodies. Anti-GFP and anti-RFP label cell bodies and axons of the eagle neurons (EG and EW). Scale bar represents 10μm (A) or 2μm (C’ and F’). (A-C’) At stage 13, Apc2 and EB1 expression co-localize in the growing axon and the cell body of the Eagle neurons. (D-F’) At stage 16, Apc2 and EB1 expression co-localize in the elongated axon of the Eagle neurons. (G-H’) Stage 15–16 embryos of the indicated genotype carrying eg-GAL4 and UAS-Apc2GFP transgenes, stained with anti-GFP antibodies. Anti-GFP labels cell bodies and axons of the eagle neurons (EG and EW). Scale bar represents 10μm (G) or 5 μm (G’). (G) and (G’) In control embryos the average of the GFP signal intensity reflecting the Apc2 transgene expression, corresponds to 89% in cell bodies and 79% in axons. (H) and (H’) *brat* homozygous mutant embryos, show a decrease of the GFP signal intensity to 50% in cell bodies and 25% in axons, reflecting a reduction of the Apc2 transgene expression. (I) Quantification of the GFP staining signal intensity shown in (G-H’). Data are presented as mean ± SEM. 10 embryos were scored for each genotype. Significance was assessed using the Student’s t-test (^∗∗∗∗^p < 0.0001).

**Fig 7:**
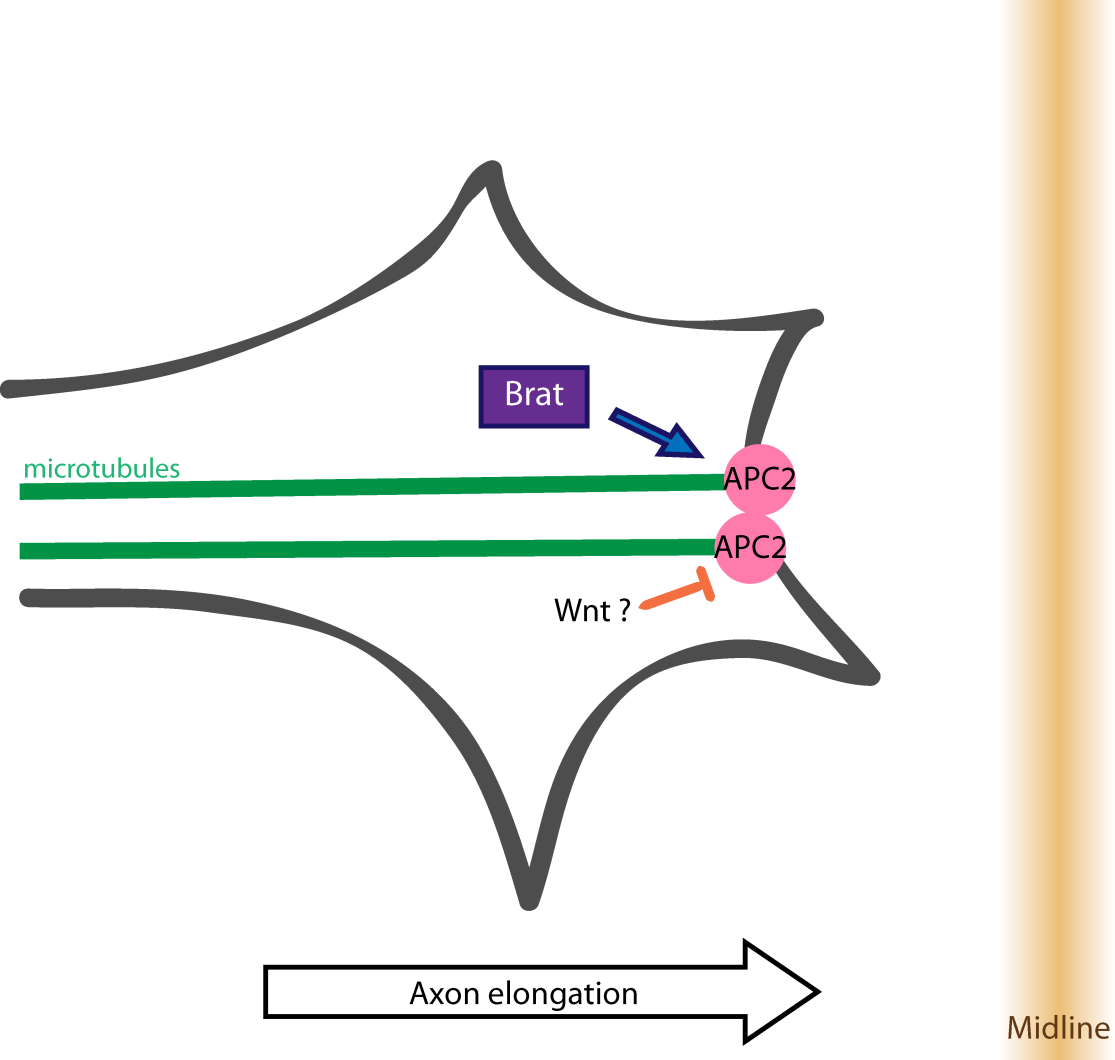
Model for how brain tumor interacts with Apc2 to promote axon growth across the midline. We propose that Brat maintains Apc2 at the plus-ends of microtubules at the periphery of the growth cone resulting in axon extension across the midline.

Taken together our findings suggest that the role of Brat in commissural axon guidance is mechanistically distinct from previous described functions of Brat in either the control of mushroom body axon maintenance or in the regulation of INP progenitor identity, although there are some shared features with the latter process. How then does Brat contribute to the growth of axons across the midline? One possibility is that rather than affecting Arm-dependent gene expression that Brat and Apc2 could influence axon growth through regulation of the neuronal cytoskeleton. Indeed, Apc2 has been shown to interact with the plus ends of microtubules and there is *in vitro* evidence that it can regulate axon growth. To test this idea, we first examined the distribution of Apc2 in wild-type Eg neurons using an Apc2-GFP fusion protein. In order to simultaneously visualize plus-ends of microtubules, we co-expressed an EB1-RFP fusion protein. Interestingly, in wild-type embryos Apc2-GFP and EB1-RFP are more clearly co-localized in stages where axons are actively growing toward the midline, relative to stages when midline crossing is complete, suggesting that Apc2 may contribute to promoting axonal growth toward the midline (Fig 6A–6F’). We next tested whether the localization or expression of Apc2 is dependent on Brat by monitoring the levels and distribution of a GFP-tagged Apc2 protein in the eagle neurons of *wild-type* and *brat* mutant embryos. Strikingly, we find that in the absence of Brat, there is a significant reduction of Apc2-GFP expression in both the cell bodies and axons of EW commissural neurons relative to heterozygous sibling controls (Fig 6G–6I). In addition, in contrast to wild-type neurons where Apc2-GFP expression appears to localize to discrete puncta, the expression of Apc2-GFP is more uniform in *brat* mutant neurons (Fig 6G and 6H). We also examined the relative levels of Brat transgene expression in *apc2* mutants but did not observe any significant difference (S5 Fig). These results support the model that Brat may promote axon guidance by maintaining the expression and localization of Apc2 to the plus-ended tips of growing microtubules during growth toward the midline (Fig 7).

## Discussion

The decision of axons to cross the midline during neural development is under complex genetic control, and multiple signaling pathways contribute to ensure that midline crossing is precisely regulated. Here we have defined a new mechanism that promotes axon growth across the midline. Through a forward genetic screen, we identified *brat* as a modulator of midline crossing and through a series of structure-function experiments and genetic analyses, we provide strong evidence that Brat function in midline crossing is distinct from its previously described roles in other developmental processes. Taken together our data support a model where Brat functions cell-autonomously in commissural neurons to promote axon growth across the midline by regulating the expression and localization of the Apc2 protein to the plus-ends of microtubules.

### Brat promotes midline crossing independently of Netrin-Fra

We identified Brat in a genetic modifier screen based on a sensitized background where Netrin-dependent axon attraction is selectively reduced in a small subset of commissural axons. Genes identified in this screen could either act in the Netrin-Fra pathway or in independent pathways to regulate midline crossing. Our genetic data indicates that Brat function is independent of Netrin-Fra, since double mutants result in significantly stronger phenotypes than *fra* or *brat* single mutants. Indeed, *brat* single mutants have no zygotic loss of function phenotype in the absence of additional perturbations to pathways that promote midline crossing, suggesting that Brat may function redundantly. There are many examples of redundant pathways that promote midline crossing in both invertebrates and in the mammalian spinal cord, whose functions are only revealed when other pathways are limited. For example, Nell2, a recently identified ligand for the Robo3 receptor, has no significant spinal commissural axon guidance phenotype unless other pro-crossing pathways are also limited [43]. Similarly, requirements for Drosophila Robo2 and Semaphorin1a to promote midline crossing are only revealed when the Netrin-Fra pathway is disrupted [14,16]. Most recently two studies made the surprising finding that floor-plate specific removal of Netrin does not result in significant midline crossing defects [44,45], suggesting that floor-plate derived Netrin may act redundantly with other pathways to promote crossing. One common interpretation of these findings is that these redundant pathways exist and are conserved to ensure robustness in the essential process of forming correct midline circuitry.

An alternative possibility that could account for the absence of a phenotype in *brat* mutants is that maternal contribution of *brat* mRNA and/or protein may be sufficient to fulfill the Brat requirement to promote crossing. There is ample precedent for maternal contribution of gene products masking requirements for embryonic axon guidance in *Drosophila*. For example, signaling components in the Robo pathway, such as Dock, Son of Sevenless, and Kuzbanian are all contributed maternally and expression is maintained throughout the entire duration of embryogenesis [46–48]. In the case of Dock, it has been shown that loss of both maternal and zygotic gene products reveals a strong phenotype, not observed in zygotic mutants [47]. The observation that simultaneous removal of zygotic expression of both Brat and Apc2 reveals a significant defect in midline crossing would lend support to the possibility that maternal gene products may be the explanation for the absence of single mutant phenotypes. Whether redundancy or maternal compensation explain the absence of *brat* and *apc2* single mutant phenotypes, our data support the interpretation that Brat and Apc2 constitute part of an important mechanism to promote axon growth across the midline.

### Brat cooperates with Apc2 to promote axon growth across the midline

Brat is a multi-domain protein with diverse functions in developing tissues. Interestingly, discrete and separable structural features of the protein control many of Brat’s distinct activities. For example, Brat’s NHL domain is required for its role as a translational repressor, but is dispensable for the control of intermediate neural progenitor identity and commissural axon guidance. Instead, these functions both appear to depend on the N-terminal B box domains of Brat, and in both axon guidance and INP specification genetic evidence points to important interactions with the Apc2 protein, a critical component of the B-catenin/Armadillo destruction complex [29], and this study. Intriguingly, in both of these cases, the loss of *brat* function results in decreased expression and altered localization of the Apc2 protein, but the role of Apc2 in these two processes appears to be distinct. Specifically, during the specification of INP identity, Apc2 acts in its classical role as a component of the destruction complex to attenuate B-catenin-dependent transcriptional activity [29]. In contrast, during commissural axon guidance, Apc2 does not regulate B-catenin-dependent transcription, but instead more likely acts locally to stabilize microtubules in the advancing growth cone. This idea is supported by our observations that the genetic manipulation of B-catenin-dependent transcription does not affect commissural axon guidance and that the enrichment of Apc2 at microtubule plus ends is diminished in *brat* mutants. In addition, previous studies in cultured vertebrate neurons support a role for Apc2 in controlling axon growth through the regulation of microtubule stability [34,35]. It will be interesting to determine whether vertebrate orthologs of Brat are also involved in these processes.

How might the activity of Brat control the specific localization of Apc2 in commissural axons to promote axon growth across the midline? One possibility is that Brat could directly associate with Apc2 and somehow deliver it to or stabilize it at microtubule plus-ends; however, no physical interactions between Brat and Apc2 have yet been detected. Alternatively, Brat may indirectly promote Apc2 localization and function through interactions with unidentified upstream signals. Based on the observation that Wnt signaling can induce the loss of Apc2 microtubule localization in vertebrate neurons [34], and that this leads to the formation of looped microtubules (a characteristic feature of paused growth cones) [49,50], it is interesting to speculate that Brat may promote continuous axon growth across the midline by stabilizing Apc2 localization at the plus-ends of microtubules. Future identification of additional components that contribute to Brat and Apc2 mediated commissural axon guidance will allow for continued dissection of the underlying cell biological mechanisms.

## Materials and methods

### Genetics

The following *Drosophila* mutant alleles were used: *fra*^*3*^, egMZ360 (eg-GAL4). The following stocks were from Bloominton: *Df(3R)Exel6198*, *Df(2L)Exel8040*, *arm*^*8*^, *UAS-arm-GFP*, *UAS-Apc2-GFP*, *UAS-arm*.*S10*, *UAS-TCF* and *UAS-ΔTCF*. The stock *Src64B-GFP* was from the Kyoto Stock Center. The following stocks were a gift from C-Y Lee: *brat*^*11*^, *UAS-brat-Myc*, *UAS-brat*^*ΔNHL*^*-Myc*, *UAS-brat*^*ΔCC*^*-Myc*, *UAS-brat*^*ΔBB*^*-Myc*, *UAS-brat*^*ΔBB1*^*-Myc*, *and UAS-brat*^*ΔBB2*^*-Myc*. The following stocks were a gift from F Besse: *UAS-brat-HA*, *UAS-brat*^*GD*^*-HA*, *and UAS-brat*^*RD*^*-HA*. The following stocks were a gift from M. Peifer: *Apc2*^*g10*^. The following transgenes were used *UAS-FraΔC-HA*, *UAS-A5CD8-GFP*. The *UAS-EB1-RFP* stock was a gift from Yuanquan Song. GAL4 drivers used were elav-GAL4 and eg-GAL4. All crosses were carried out at 25°C. Embryos were genotyped using balancer chromosomes carrying lacZ markers or by the presence of epitope-tagged transgenes. See S1 Table for a complete list of genotypes for all the figures.

### Immunofluorescence

Dechorionated, formaldehyde-fixed, methanol devitellinized embryos were fluorescently stained as previously described [51]. The following primary antibodies were used: mouse anti-1D4/FasII [Developmental Studies Hybridoma Bank (DSHB); 1:100], mouse anti-Beta gal [DSHB; 1:150], mouse anti-Myc [DSHB (9E10); 1:500] rabbit anti-GFP [Invitrogen (#A11122); 1:500], Mouse anti-HA [Covance (16B12) 1:250], Chicken anti-GFP [Aves Labs (GFP-1020) 1:1000]. The following secondary antibodies were used: Alexa647- conjugated goat anti-HRP [Jackson Immunoresearch (#123-605-021); 1:500]. Cyanine 3-conjugated goat anti-rabbit [Jackson; 1:1000], Alexa488-conjugated goat anti-mouse [Molecular Probes; 1:500] and Alexa488-conjugated donkey anti-chicken [Jackson Immunoresearch; 1:500]. Embryos were mounted in 70% glycerol/PBS.

### *In Situ* Hybridization

Fluorescent mRNA in situ hybridization was performed as described, with digoxigenin labeled probes [52]. Briefly, hybridized probe was detected with anti-digoxigenin-HRP (Roche), using fluorescein-labeled tyramide as a substrate (TSA Fluorescence System, Perkin Elmer). Embryos were mounted in 70% glycerol/PBS.

### Imaging

Phenotypes were analyzed and images were acquired using a spinning disk confocal system (Perkin Elmer) built on a Nikon Ti-U inverted microscope using a Nikon OFN25 60x 40x or 10x objective with a Hamamatsu C10600-10B CCD camera and Yokogawa CSU-10 scanner head with Volocity imaging software. Images were processed using ImageJ and Adobe Illustrator software. For fluorescence quantification of GFP antibody staining in embryos, ten embryos per genotype (+/+; *UAS-Apc2GFP* or *brat(-); UAS-Apc2GFP*, *+/+; src64bGFP or brat(-); scr64bGFP*) were imaged using identical settings. Max projections were generated using ImageJ. After subtracting the staining background, the average pixel intensity was measured on twelve to sixteen clusters of EW neurons or across five regions within longitudinal axons for each embryo. The values from the five to ten embryos for each phenotype were averaged.

### Phenotypic quantification

For EW commissural neuron axon crossing phenotypes, whole-mount or filleted embryos were analyzed at Stages 15 and 16. Eight abdominal segments were analyzed per embryo when possible, and for each embryo, the percentage of non-crossing segments was calculated. A segment was considered non-crossing when both clusters of EW axons (six axons per segment) failed to reach the midline. Embryos were scored blind to genotype when possible.

### Statistics

For statistical analysis, comparisons were made between genotypes using the Student’s t-test, ANOVA or Chi-squared test. For multiple comparisons, significance was assessed by using a Bonferroni correction.

## Supporting information

S1 Fig *brat* mRNA is expressed in the central nervous system during axon midline crossing. (A-J’) Stage 13–17 embryos of the indicated genotypes carrying eg-GAL4 and UAS-tauMycGFP transgenes, stained with anti-DIG (green) (A-J’) and anti-GFP (magenta). Anti-DIG reveals *brat* mRNA, Anti-GFP labels cell bodies and axons of the eagle neurons (EG and EW). Scale bar represents 10μm (A, F), 5μm (F’). (A-E) In whole mount embryos, *brat* mRNA (in green) is detected in the ventral nerve cord and the brain during all the stages of development (Stages 13 to 17), when axons grow and cross the midline. (F-J’) Dissected embryos reveal that *brat* mRNA (in green) is expressed in Eagle neurons (magenta) during stage 13 to 17.
(TIF)

S2 Fig *Brat* over-expression suppresses the FraΔC effect on midline crossing. (A-B’) Stage 15–16 embryos of the indicated genotype carrying the elav-GAL4 transgene, stained with anti-FasII (green) (A-B) and anti-Myc (red) (A’-B’) antibodies. Anti-FasII labels the ipsilateral axons, anti-Myc reveals the UAS-Brat transgene expression. Scale bar represents 10μm (A) and 5μm (A’). (C-D) Stage 15–16 embryos of the indicated genotype carrying eg-GAL4 and UAS-FraΔC transgenes, stained with anti-GFP antibodies. Anti-GFP labels cell bodies and axons of the eagle neurons (EG and EW). Scale bar represents 10μm (C). (A) In wild-type embryos Fas2 positive ipsilateral axons turn before reaching the midline to grow longitudinally in all segments. (B) Expressing UAS-Brat in all neurons does not induce any ectopic crossing of ipsilateral axons. (C) EW axons fail to cross in 27% of segments when UAS-FraΔC is selectively expressed in eagle neurons. (D) In the FraΔC background the expression of UAS-Brat in eagle neurons reduces the EW crossing defects to 17%. (E) Quantification of EW midline crossing defects in the genotypes shown in (C-D). Data are presented as mean ± SEM. 20 embryos were scored for each genotype. Significance was assessed using the Student’s t-test (p<0.05).
(TIF)

S3 Fig Expression of different UAS-Brat HA or Myc-tagged transgenes in the Eagle neurons. (A-I) Stage 15–16 embryos of the indicated genotype carrying eg-GAL4 and UAS-BratHA (A), UAS-Brat^GD^HA (B), UAS-Brat^RD^HA (C), UAS-Bratmyc (D), UAS-Brat^NHL^Myc (E), UAS-Brat^CC^Myc (F), UAS-Brat^BB^Myc (G), UAS-Brat^BB1^Myc (H) or UAS-Brat^BB2^Myc (I) transgenes, stained with anti-HA (A-C) or anti-Myc (D-I) (green) and anti-HRP (blue (A-C) or magenta (D-I)) antibodies. Anti-HA and Anti-Myc labels cell bodies and axons of the eagle neurons (EG and EW), Anti-HRP reveals all of the CNS axons. Scale bar represents 10μm (A and D). (A-I) When driven by the eg-GAL4 transgene, the three UAS-Brat tagged HA and the six UAS-Brat tagged Myc transgenes are expressed at similar levels in the cell bodies and axons during the studied development stages.
(TIF)

S4 Fig The expression of Src64b is not perturbed in *brat* null mutant embryos. (A-F’) Stage 14–17 embryos of the indicated genotype, stained with anti-GFP (green) and anti-HRP (magenta) antibodies. Anti-GFP labels the fusion protein Src-GFP, Anti-HRP reveals all of the CNS axons. Scale bar represents 10μm (A). (A-F) Src-GFP is expressed in all neurons from stage 13 to 17. (A-C) In *wild type* embryos, the average of the GFP signal intensity, reflecting Src64b expression, corresponds to 73%. (D-F) In *brat* mutant embryos, the GFP signal remains the same intensity compare to *wild type* embryos (70%). (G) Quantification of the GFP staining signal intensity shown in (A-F). Data are presented as mean ± SEM. 10 embryos were scored for each genotype. Significance was assessed using the Student’s t-test (ns, p > 0.05).
(TIF)

S5 Fig The expression of the transgene UAS-BratHA is not changed in *Apc2* null mutant embryos. (A-B’) Stage 15–16 embryos of the indicated genotype carrying eg-GAL4 and UAS-bratHA transgenes, stained with anti-HA antibodies. Anti-HA labels cell bodies of the eagle neurons (EG and EW). (A) and (A’) In control embryos the average of the HA signal intensity reflecting the Brat transgene expression, corresponds to 71% in cell bodies. (B) and (B’) *Apc2* homozygous mutant embryos, show a similar HA signal intensity in cell bodies (73%), the absence of *Apc2* does not perturb the Brat transgene expression. (B) Quantification of the HA staining signal intensity shown in (A-B’). Data are presented as mean ± SEM. 3 embryos were scored for each genotype. Significance was assessed using the Student’s t-test (ns, p > 0.05)
(TIF)

S1 Table List of the genotypes analyzed in each figure. Related to Figs 1–6 and S1–S5 Figs. The table lists the full genotypes that correspond to the abbreviated genotypes presented in the main and supplemental figures. Please see associated Microsoft Excel spreadsheet.
(PDF)

## References

[1] Neuhaus-Follini A, Bashaw GJ (2015) Crossing the embryonic midline: molecular mechanisms regulating axon responsiveness at an intermediate target. Wiley Interdiscip Rev Dev Biol. 10.1002/wdev.185 PMC4456295 25779002

[2] Bashaw GJ, Klein R (2010) Signaling from axon guidance receptors. Cold Spring Harb Perspect Biol 2: a001941 doi: 10.1101/cshperspect.a001941 20452961 10.1101/cshperspect.a001941 PMC2857166

[3] Serafini T, Kennedy TE, Galko MJ, Mirzayan C, Jessell TM, et al (1994) The netrins define a family of axon outgrowth-promoting proteins homologous to C. elegans UNC-6. Cell 78: 409–424. 8062384 10.1016/0092-8674(94)90420-0

[4] Kennedy TE, Serafini T, de la Torre JR, Tessier-Lavigne M (1994) Netrins are diffusible chemotropic factors for commissural axons in the embryonic spinal cord. Cell 78: 425–435. 8062385 10.1016/0092-8674(94)90421-9

[5] Kolodziej PA, Timpe LC, Mitchell KJ, Fried SR, Goodman CS, et al (1996) frazzled encodes a Drosophila member of the DCC immunoglobulin subfamily and is required for CNS and motor axon guidance. Cell 87: 197–204. 8861904 10.1016/s0092-8674(00)81338-0

[6] Marsh AP, Heron D, Edwards TJ, Quartier A, Galea C, et al (2017) Mutations in DCC cause isolated agenesis of the corpus callosum with incomplete penetrance. Nat Genet 49: 511–514. doi: 10.1038/ng.3794 28250454 10.1038/ng.3794 PMC5894478

[7] Srour M, Riviere JB, Pham JM, Dube MP, Girard S, et al (2010) Mutations in DCC cause congenital mirror movements. Science 328: 592 doi: 10.1126/science.1186463 20431009 10.1126/science.1186463

[8] Rabe Bernhardt N, Memic F, Gezelius H, Thiebes AL, Vallstedt A, et al (2012) DCC mediated axon guidance of spinal interneurons is essential for normal locomotor central pattern generator function. Dev Biol 366: 279–289. doi: 10.1016/j.ydbio.2012.03.017 22521513 10.1016/j.ydbio.2012.03.017

[9] Blockus H, Chedotal A (2014) The multifaceted roles of Slits and Robos in cortical circuits: from proliferation to axon guidance and neurological diseases. Curr Opin Neurobiol 27: 82–88. doi: 10.1016/j.conb.2014.03.003 24698714 10.1016/j.conb.2014.03.003

[10] Jamuar SS, Schmitz-Abe K, D'Gama AM, Drottar M, Chan WM, et al (2017) Biallelic mutations in human DCC cause developmental split-brain syndrome. Nat Genet 49: 606–612. doi: 10.1038/ng.3804 28250456 10.1038/ng.3804 PMC5374027

[11] Garbe DS, O'Donnell M, Bashaw GJ (2007) Cytoplasmic domain requirements for Frazzled-mediated attractive axon turning at the Drosophila midline. Development 134: 4325–4334. doi: 10.1242/dev.012872 18003737 10.1242/dev.012872

[12] Mitchell KJ, Doyle JL, Serafini T, Kennedy TE, Tessier-Lavigne M, et al (1996) Genetic analysis of Netrin genes in Drosophila: Netrins guide CNS commissural axons and peripheral motor axons. Neuron 17: 203–215. 8780645 10.1016/s0896-6273(00)80153-1

[13] Charron F, Stein E, Jeong J, McMahon AP, Tessier-Lavigne M (2003) The morphogen sonic hedgehog is an axonal chemoattractant that collaborates with netrin-1 in midline axon guidance. Cell 113: 11–23. 12679031 10.1016/s0092-8674(03)00199-5

[14] Hernandez-Fleming M, Rohrbach EW, Bashaw GJ (2017) Sema-1a Reverse Signaling Promotes Midline Crossing in Response to Secreted Semaphorins. Cell Rep 18: 174–184. doi: 10.1016/j.celrep.2016.12.027 28052247 10.1016/j.celrep.2016.12.027 PMC5253228

[15] Ruiz de Almodovar C, Coulon C, Salin PA, Knevels E, Chounlamountri N, et al (2010) Matrix-binding vascular endothelial growth factor (VEGF) isoforms guide granule cell migration in the cerebellum via VEGF receptor Flk1. J Neurosci 30: 15052–15066. doi: 10.1523/JNEUROSCI.0477-10.2010 21068311 10.1523/JNEUROSCI.0477-10.2010 PMC6633861

[16] Evans TA, Santiago C, Arbeille E, Bashaw GJ (2015) Robo2 acts in trans to inhibit Slit-Robo1 repulsion in pre-crossing commissural axons. Elife 4: e08407 doi: 10.7554/eLife.08407 26186094 10.7554/eLife.08407 PMC4505356

[17] Hernandez-Enriquez B, Wu Z, Martinez E, Olsen O, Kaprielian Z, et al (2015) Floor plate-derived neuropilin-2 functions as a secreted semaphorin sink to facilitate commissural axon midline crossing. Genes Dev 29: 2617–2632. doi: 10.1101/gad.268086.115 26680304 10.1101/gad.268086.115 PMC4699389

[18] Nawabi H, Briancon-Marjollet A, Clark C, Sanyas I, Takamatsu H, et al (2010) A midline switch of receptor processing regulates commissural axon guidance in vertebrates. Genes Dev 24: 396–410. doi: 10.1101/gad.542510 20159958 10.1101/gad.542510 PMC2816738

[19] Neuhaus-Follini A, Bashaw GJ (2015) The Intracellular Domain of the Frazzled/DCC Receptor Is a Transcription Factor Required for Commissural Axon Guidance. Neuron 87: 751–763. doi: 10.1016/j.neuron.2015.08.006 26291159 10.1016/j.neuron.2015.08.006 PMC4754793

[20] Zou Y, Stoeckli E, Chen H, Tessier-Lavigne M (2000) Squeezing axons out of the gray matter: a role for slit and semaphorin proteins from midline and ventral spinal cord. Cell 102: 363–375. 10975526 10.1016/s0092-8674(00)00041-6

[21] O'Donnell MP, Bashaw GJ (2013) Distinct functional domains of the Abelson tyrosine kinase control axon guidance responses to Netrin and Slit to regulate the assembly of neural circuits. Development 140: 2724–2733. doi: 10.1242/dev.093831 23720041 10.1242/dev.093831 PMC3678342

[22] Arama E, Dickman D, Kimchie Z, Shearn A, Lev Z (2000) Mutations in the beta-propeller domain of the Drosophila brain tumor (brat) protein induce neoplasm in the larval brain. Oncogene 19: 3706–3716. doi: 10.1038/sj.onc.1203706 10949924 10.1038/sj.onc.1203706

[23] Sonoda J, Wharton RP (2001) Drosophila Brain Tumor is a translational repressor. Genes Dev 15: 762–773. doi: 10.1101/gad.870801 11274060 10.1101/gad.870801 PMC312658

[24] Wulczyn FG, Cuevas E, Franzoni E, Rybak A (2011) miRNAs Need a Trim: Regulation of miRNA Activity by Trim-NHL Proteins. Adv Exp Med Biol 700: 85–105. doi: 10.1007/978-1-4419-7823-3_9 21755476 10.1007/978-1-4419-7823-3_9

[25] Neumuller RA, Betschinger J, Fischer A, Bushati N, Poernbacher I, et al (2008) Mei-P26 regulates microRNAs and cell growth in the Drosophila ovarian stem cell lineage. Nature 454: 241–245. doi: 10.1038/nature07014 18528333 10.1038/nature07014 PMC2988194

[26] Bowman SK, Rolland V, Betschinger J, Kinsey KA, Emery G, et al (2008) The tumor suppressors Brat and Numb regulate transit-amplifying neuroblast lineages in Drosophila. Dev Cell 14: 535–546. doi: 10.1016/j.devcel.2008.03.004 18342578 10.1016/j.devcel.2008.03.004 PMC2988195

[27] Edwards TA, Wilkinson BD, Wharton RP, Aggarwal AK (2003) Model of the brain tumor-Pumilio translation repressor complex. Genes Dev 17: 2508–2513. doi: 10.1101/gad.1119403 14561773 10.1101/gad.1119403 PMC218144

[28] Marchetti G, Reichardt I, Knoblich JA, Besse F (2014) The TRIM-NHL protein Brat promotes axon maintenance by repressing src64B expression. J Neurosci 34: 13855–13864. doi: 10.1523/JNEUROSCI.3285-13.2014 25297111 10.1523/JNEUROSCI.3285-13.2014 PMC6608379

[29] Komori H, Xiao Q, McCartney BM, Lee CY (2014) Brain tumor specifies intermediate progenitor cell identity by attenuating beta-catenin/Armadillo activity. Development 141: 51–62. doi: 10.1242/dev.099382 24257623 10.1242/dev.099382 PMC3865749

[30] Bienz M (2002) The subcellular destinations of APC proteins. Nat Rev Mol Cell Biol 3: 328–338. doi: 10.1038/nrm806 11988767 10.1038/nrm806

[31] Mattie FJ, Stackpole MM, Stone MC, Clippard JR, Rudnick DA, et al (2010) Directed microtubule growth, +TIPs, and kinesin-2 are required for uniform microtubule polarity in dendrites. Curr Biol 20: 2169–2177. doi: 10.1016/j.cub.2010.11.050 21145742 10.1016/j.cub.2010.11.050 PMC3035180

[32] McCartney BM, Dierick HA, Kirkpatrick C, Moline MM, Baas A, et al (1999) Drosophila APC2 is a cytoskeletally-associated protein that regulates wingless signaling in the embryonic epidermis. J Cell Biol 146: 1303–1318. 10491393 10.1083/jcb.146.6.1303 PMC2156123

[33] van Es JH, Kirkpatrick C, van de Wetering M, Molenaar M, Miles A, et al (1999) Identification of APC2, a homologue of the adenomatous polyposis coli tumour suppressor. Curr Biol 9: 105–108. 10021369 10.1016/s0960-9822(99)80024-4

[34] Purro SA, Ciani L, Hoyos-Flight M, Stamatakou E, Siomou E, et al (2008) Wnt regulates axon behavior through changes in microtubule growth directionality: a new role for adenomatous polyposis coli. J Neurosci 28: 8644–8654. doi: 10.1523/JNEUROSCI.2320-08.2008 18716223 10.1523/JNEUROSCI.2320-08.2008 PMC2832753

[35] Shintani T, Ihara M, Tani S, Sakuraba J, Sakuta H, et al (2009) APC2 plays an essential role in axonal projections through the regulation of microtubule stability. J Neurosci 29: 11628–11640. doi: 10.1523/JNEUROSCI.2394-09.2009 19759310 10.1523/JNEUROSCI.2394-09.2009 PMC6665762

[36] Higashijima S, Shishido E, Matsuzaki M, Saigo K (1996) eagle, a member of the steroid receptor gene superfamily, is expressed in a subset of neuroblasts and regulates the fate of their putative progeny in the Drosophila CNS. Development 122: 527–536. 8625804 10.1242/dev.122.2.527

[37] Cho PF, Gamberi C, Cho-Park YA, Cho-Park IB, Lasko P, et al (2006) Cap-dependent translational inhibition establishes two opposing morphogen gradients in Drosophila embryos. Curr Biol 16: 2035–2041. doi: 10.1016/j.cub.2006.08.093 17055983 10.1016/j.cub.2006.08.093 PMC2238800

[38] Olesnicky EC, Bhogal B, Gavis ER (2012) Combinatorial use of translational co-factors for cell type-specific regulation during neuronal morphogenesis in Drosophila. Dev Biol 365: 208–218. doi: 10.1016/j.ydbio.2012.02.028 22391052 10.1016/j.ydbio.2012.02.028 PMC3642870

[39] Loedige I, Stotz M, Qamar S, Kramer K, Hennig J, et al (2014) The NHL domain of BRAT is an RNA-binding domain that directly contacts the hunchback mRNA for regulation. Genes Dev 28: 749–764. doi: 10.1101/gad.236513.113 24696456 10.1101/gad.236513.113 PMC4015489

[40] Harris RE, Pargett M, Sutcliffe C, Umulis D, Ashe HL (2011) Brat promotes stem cell differentiation via control of a bistable switch that restricts BMP signaling. Dev Cell 20: 72–83. doi: 10.1016/j.devcel.2010.11.019 21238926 10.1016/j.devcel.2010.11.019 PMC3178012

[41] O'Donnell MP, Bashaw GJ (2013) Src Inhibits Midline Axon Crossing Independent of Frazzled/Deleted in Colorectal Carcinoma (DCC) Receptor Tyrosine Phosphorylation. J Neurosci 33: 305–314. doi: 10.1523/JNEUROSCI.2756-12.2013 23283343 10.1523/JNEUROSCI.2756-12.2013 PMC3739878

[42] Pai LM, Orsulic S, Bejsovec A, Peifer M (1997) Negative regulation of Armadillo, a Wingless effector in Drosophila. Development 124: 2255–2266. 9187151 10.1242/dev.124.11.2255

[43] Jaworski A, Tom I, Tong RK, Gildea HK, Koch AW, et al (2015) Operational redundancy in axon guidance through the multifunctional receptor Robo3 and its ligand NELL2. Science 350: 961–965. doi: 10.1126/science.aad2615 26586761 10.1126/science.aad2615

[44] Dominici C, Moreno-Bravo JA, Puiggros SR, Rappeneau Q, Rama N, et al (2017) Floor-plate-derived netrin-1 is dispensable for commissural axon guidance. Nature 545: 350–354. doi: 10.1038/nature22331 28445456 10.1038/nature22331 PMC5438598

[45] Varadarajan SG, Kong JH, Phan KD, Kao TJ, Panaitof SC, et al (2017) Netrin1 Produced by Neural Progenitors, Not Floor Plate Cells, Is Required for Axon Guidance in the Spinal Cord. Neuron 94: 790–799 e793. doi: 10.1016/j.neuron.2017.03.007 28434801 10.1016/j.neuron.2017.03.007 PMC5576449

[46] Coleman HA, Labrador JP, Chance RK, Bashaw GJ (2010) The Adam family metalloprotease Kuzbanian regulates the cleavage of the roundabout receptor to control axon repulsion at the midline. Development 137: 2417–2426. doi: 10.1242/dev.047993 20570941 10.1242/dev.047993 PMC2889607

[47] Fan X, Labrador JP, Hing H, Bashaw GJ (2003) Slit stimulation recruits Dock and Pak to the roundabout receptor and increases Rac activity to regulate axon repulsion at the CNS midline. Neuron 40: 113–127. 14527437 10.1016/s0896-6273(03)00591-9

[48] Yang L, Bashaw GJ (2006) Son of sevenless directly links the Robo receptor to rac activation to control axon repulsion at the midline. Neuron 52: 595–607. doi: 10.1016/j.neuron.2006.09.039 17114045 10.1016/j.neuron.2006.09.039

[49] Ciani L, Krylova O, Smalley MJ, Dale TC, Salinas PC (2004) A divergent canonical WNT-signaling pathway regulates microtubule dynamics: dishevelled signals locally to stabilize microtubules. J Cell Biol 164: 243–253. doi: 10.1083/jcb.200309096 14734535 10.1083/jcb.200309096 PMC2172322

[50] Salinas PC (2007) Modulation of the microtubule cytoskeleton: a role for a divergent canonical Wnt pathway. Trends Cell Biol 17: 333–342. doi: 10.1016/j.tcb.2007.07.003 17643305 10.1016/j.tcb.2007.07.003

[51] Bashaw GJ (2010) Visualizing axons in the Drosophila central nervous system using immunohistochemistry and immunofluorescence. Cold Spring Harb Protoc 2010: pdb prot5503. 10.1101/pdb.prot5503 PMC4765902 20889700

[52] Yang L, Garbe DS, Bashaw GJ (2009) A frazzled/DCC-dependent transcriptional switch regulates midline axon guidance. Science 324: 944–947. doi: 10.1126/science.1171320 19325078 10.1126/science.1171320 PMC4078765

