# Supplementary figures and images for: Brain Tumor promotes axon growth across the midline through interactions with the microtubule stabilizing protein Apc2
Source: PLoS Genet. 2018 Apr 4;14(4):e1007314. doi: 10.1371/journal.pgen.1007314 (PMC5902039; doi:10.1371/journal.pgen.1007314)

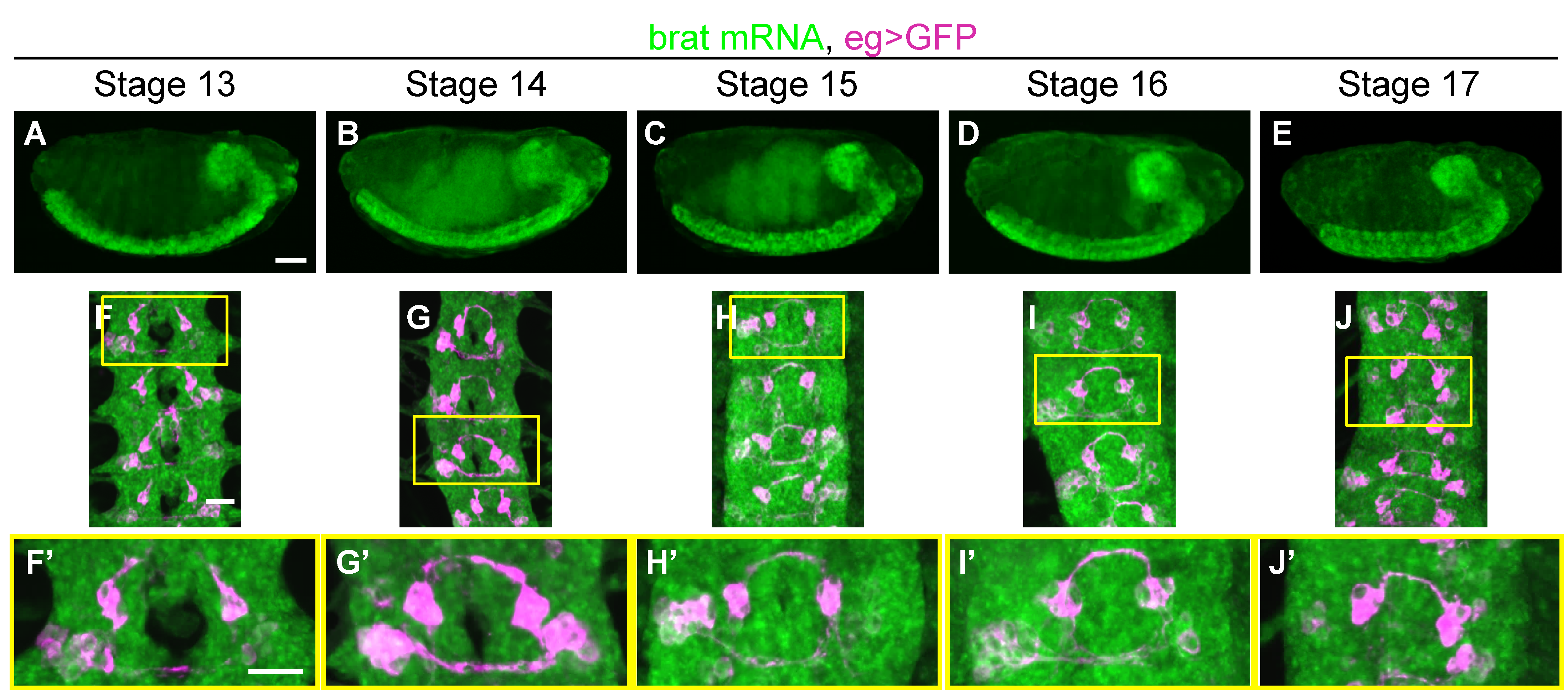

Supplement: S1 Fig — (A-J’) Stage 13–17 embryos of the indicated genotypes carrying eg-GAL4 and UAS-tauMycGFP transgenes, stained with anti-DIG (green) (A-J’) and anti-GFP (magenta). Anti-DIG reveals brat mRNA, Anti-GFP labels cell bodies and axons of the eagle neurons (EG and EW). Scale bar represents 10μm (A, F), 5μm (F’). (A-E) In whole mount embryos, brat mRNA (in green) is detected in the ventral nerve cord and the brain during all the stages of development (Stages 13 to 17), when axons grow and cross the midline. (F-J’) Dissected embryos reveal that brat mRNA (in green) is expressed in Eagle neurons (magenta) during stage 13 to 17. (TIF) [file pgen.1007314.s001.tif]

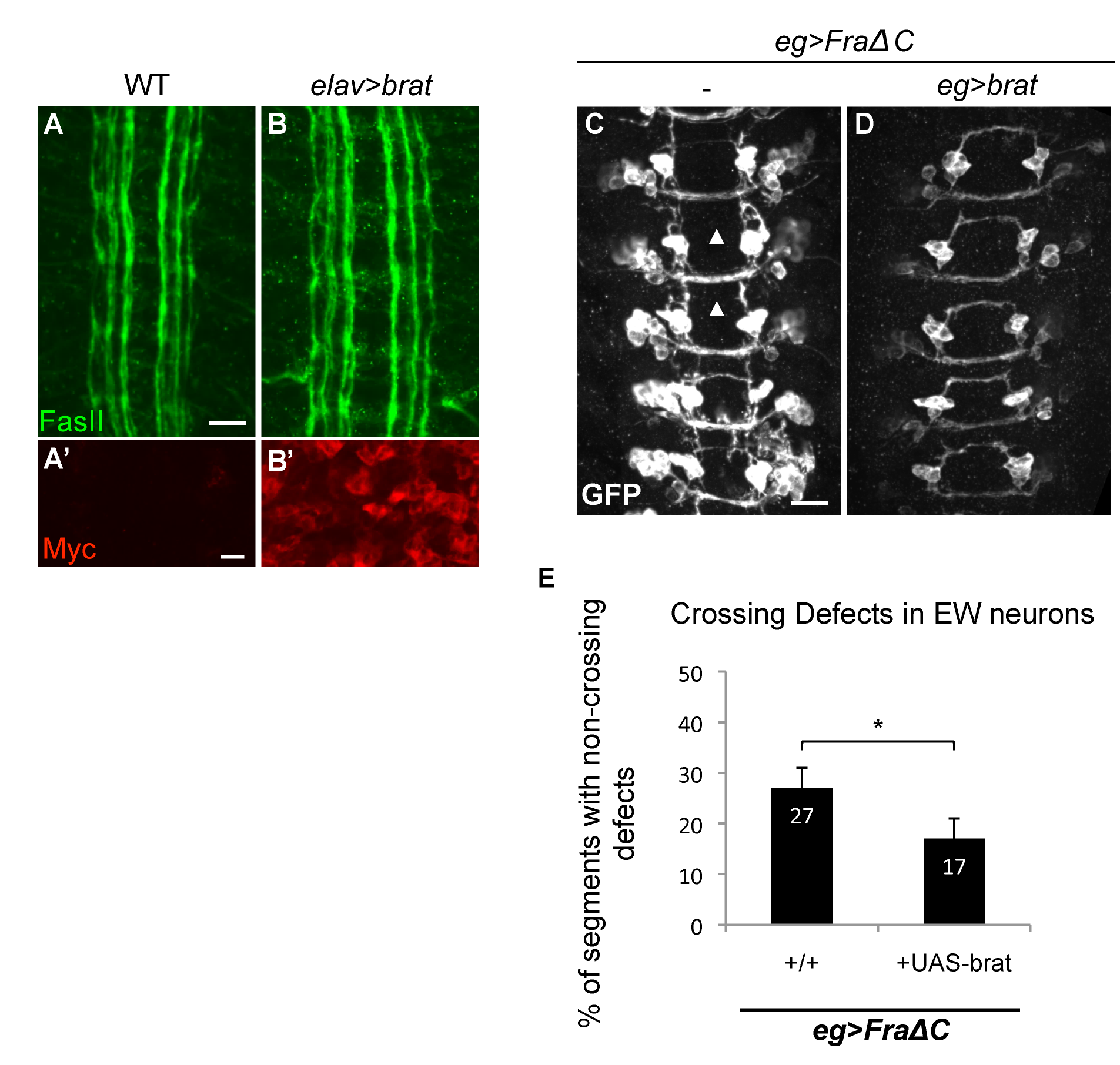

Supplement: S2 Fig — (A-B’) Stage 15–16 embryos of the indicated genotype carrying the elav-GAL4 transgene, stained with anti-FasII (green) (A-B) and anti-Myc (red) (A’-B’) antibodies. Anti-FasII labels the ipsilateral axons, anti-Myc reveals the UAS-Brat transgene expression. Scale bar represents 10μm (A) and 5μm (A’). (C-D) Stage 15–16 embryos of the indicated genotype carrying eg-GAL4 and UAS-FraΔC transgenes, stained with anti-GFP antibodies. Anti-GFP labels cell bodies and axons of the eagle neurons (EG and EW). Scale bar represents 10μm (C). (A) In wild-type embryos Fas2 positive ipsilateral axons turn before reaching the midline to grow longitudinally in all segments. (B) Expressing UAS-Brat in all neurons does not induce any ectopic crossing of ipsilateral axons. (C) EW axons fail to cross in 27% of segments when UAS-FraΔC is selectively expressed in eagle neurons. (D) In the FraΔC background the expression of UAS-Brat in eagle neurons reduces the EW crossing defects to 17%. (E) Quantification of EW midline crossing defects in the genotypes shown in (C-D). Data are presented as mean ± SEM. 20 embryos were scored for each genotype. Significance was assessed using the Student’s t-test (p<0.05). (TIF) [file pgen.1007314.s002.tif]

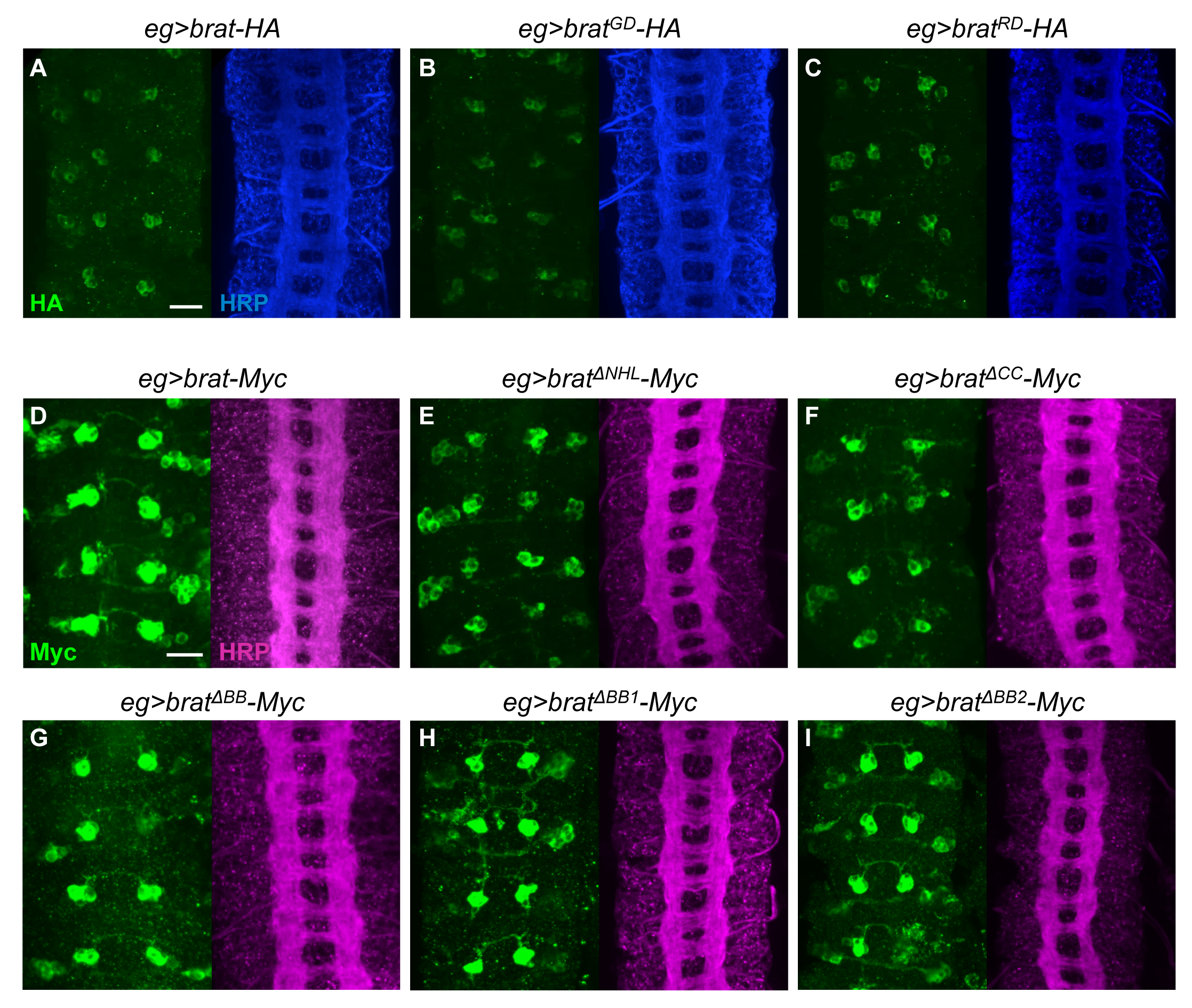

Supplement: S3 Fig — (A-I) Stage 15–16 embryos of the indicated genotype carrying eg-GAL4 and UAS-BratHA (A), UAS-BratGDHA (B), UAS-BratRDHA (C), UAS-Bratmyc (D), UAS-BratNHLMyc (E), UAS-BratCCMyc (F), UAS-BratBBMyc (G), UAS-BratBB1Myc (H) or UAS-BratBB2Myc (I) transgenes, stained with anti-HA (A-C) or anti-Myc (D-I) (green) and anti-HRP (blue (A-C) or magenta (D-I)) antibodies. Anti-HA and Anti-Myc labels cell bodies and axons of the eagle neurons (EG and EW), Anti-HRP reveals all of the CNS axons. Scale bar represents 10μm (A and D). (A-I) When driven by the eg-GAL4 transgene, the three UAS-Brat tagged HA and the six UAS-Brat tagged Myc transgenes are expressed at similar levels in the cell bodies and axons during the studied development stages. (TIF) [file pgen.1007314.s003.tif]

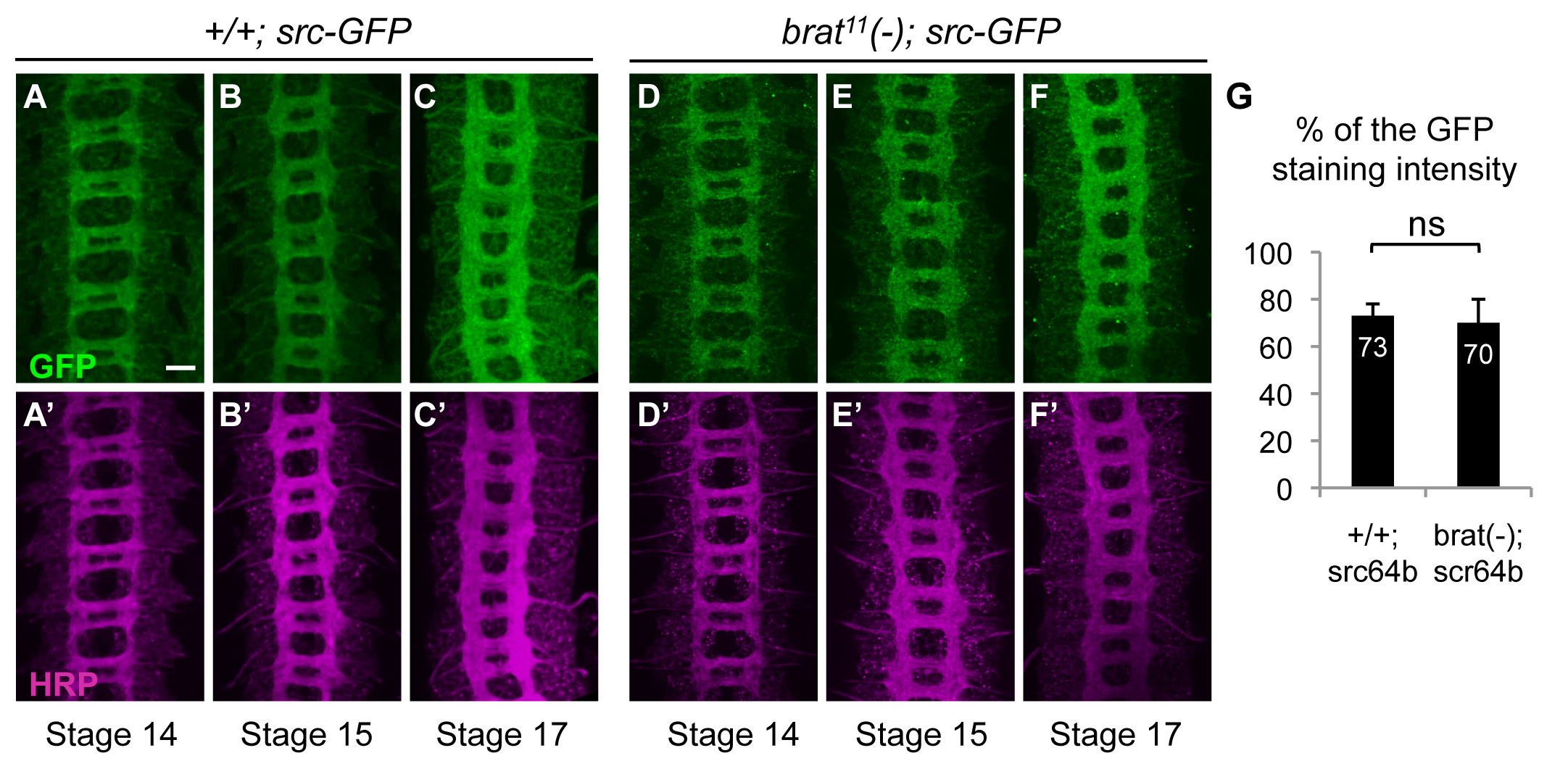

Supplement: S4 Fig — (A-F’) Stage 14–17 embryos of the indicated genotype, stained with anti-GFP (green) and anti-HRP (magenta) antibodies. Anti-GFP labels the fusion protein Src-GFP, Anti-HRP reveals all of the CNS axons. Scale bar represents 10μm (A). (A-F) Src-GFP is expressed in all neurons from stage 13 to 17. (A-C) In wild type embryos, the average of the GFP signal intensity, reflecting Src64b expression, corresponds to 73%. (D-F) In brat mutant embryos, the GFP signal remains the same intensity compare to wild type embryos (70%). (G) Quantification of the GFP staining signal intensity shown in (A-F). Data are presented as mean ± SEM. 10 embryos were scored for each genotype. Significance was assessed using the Student’s t-test (ns, p > 0.05). (TIF) [file pgen.1007314.s004.tif]

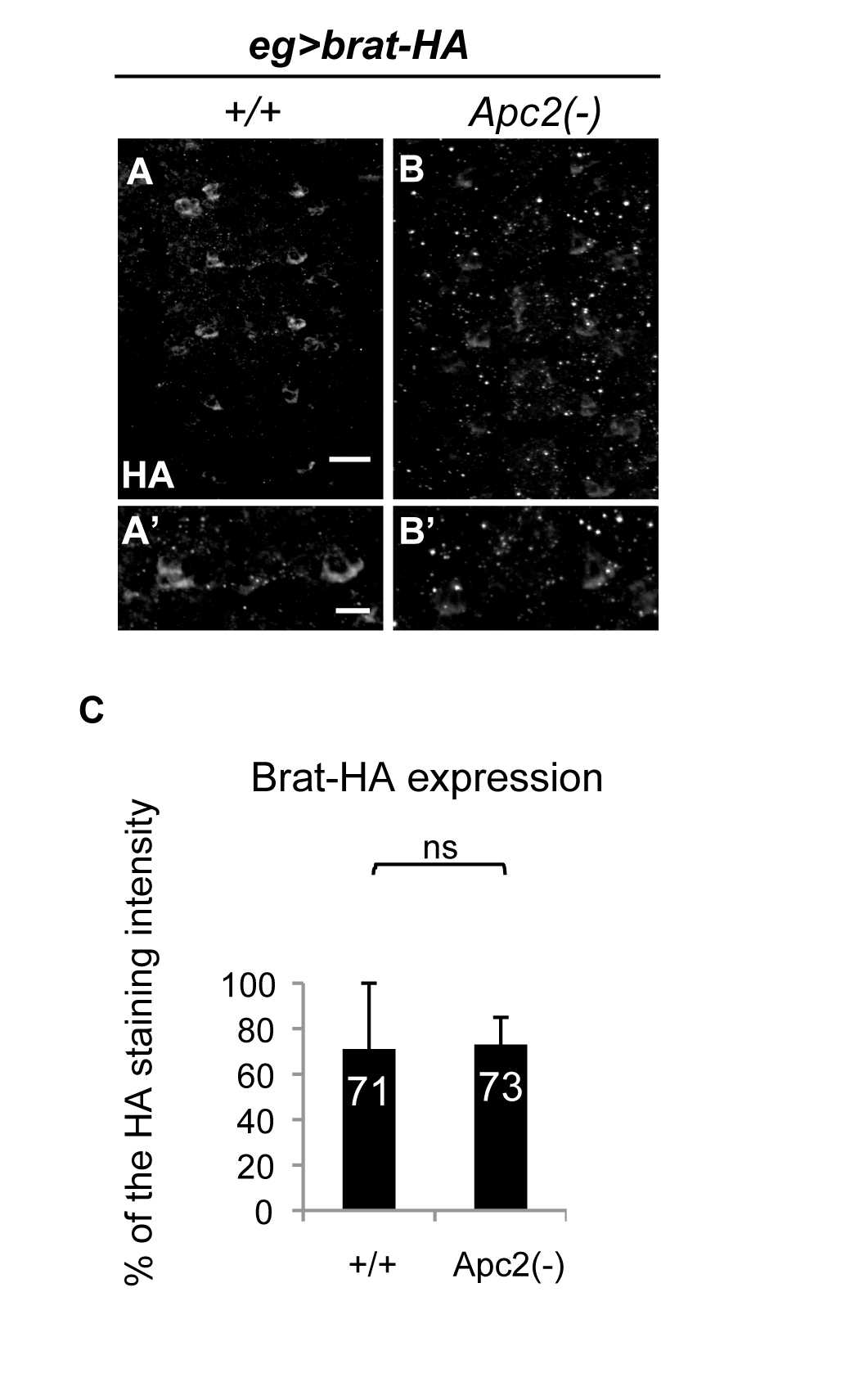

Supplement: S5 Fig — (A-B’) Stage 15–16 embryos of the indicated genotype carrying eg-GAL4 and UAS-bratHA transgenes, stained with anti-HA antibodies. Anti-HA labels cell bodies of the eagle neurons (EG and EW). (A) and (A’) In control embryos the average of the HA signal intensity reflecting the Brat transgene expression, corresponds to 71% in cell bodies. (B) and (B’) Apc2 homozygous mutant embryos, show a similar HA signal intensity in cell bodies (73%), the absence of Apc2 does not perturb the Brat transgene expression. (B) Quantification of the HA staining signal intensity shown in (A-B’). Data are presented as mean ± SEM. 3 embryos were scored for each genotype. Significance was assessed using the Student’s t-test (ns, p > 0.05) (TIF) [file pgen.1007314.s005.tif]
