# Supplementary material for: Brain Tumor promotes axon growth across the midline through interactions with the microtubule stabilizing protein Apc2
Source: PLoS Genet. 2018 Apr 4;14(4):e1007314. doi: 10.1371/journal.pgen.1007314 (PMC5902039; doi:10.1371/journal.pgen.1007314)
Supplement: S1 Table — Related to Figs 1–6 and S1–S5 Figs. The table lists the full genotypes that correspond to the abbreviated genotypes presented in the main and supplemental figures. Please see associated Microsoft Excel spreadsheet. (PDF) [file pgen.1007314.s006.pdf]

Figure	Label	Genotype
Figure 1	wild type	UAS-tauMycGFP/+; egGal4/+
	frazzled(-)	fra^{3},UAS-tauMycGFP/fra^{3}; egGal4/+
	eg>FraΔC	UAS-tauMycGFP/+; egGal4, UAS-FraΔCHA/+
	Df(2L)Exel8040 eg>FraΔC	Df(2L)Exel8040/UAS-tauMycGFP; egGal4, UAS-FraΔCHA/+
	brat/+ eg>FraΔC	brat^{11},UAS-tauMycGFP/+; egGal4, UAS-FraΔCHA/+
	brat(-) eg>FraΔC	brat^{11},UAS-tauMycGFP/brat^{11}; egGal4, UAS-FraΔCHA/+
	brat/+ + UAS-brat eg>FraΔC	brat^{11},UAS-tauMycGFP/+; egGal4, UAS-FraΔCHA/UAS-bratMyc
	brat(-)+ UAS-brat eg>FraΔC	brat^{11},UAS-tauMycGFP/brat^{11}; egGal4, UAS-FraΔCHA/UAS-bratMyc
Figure 2	wild type	UAS-tauMycGFP/+; egGal4/+
	fra(-)	fra^{3},UAS-tauMycGFP/fra^{3}; egGal4/+
	brat(-)	brat^{11},UAS-tauMycGFP/brat^{11}; egGal4/+
	fra(-), brat(-)	fra^{3},brat^{11},UAS-tauMycGFP/ fra3,brat^{11}; egGal4/+
	fra(-), brat(-)+UAS-brat	fra^{3},brat^{11}, UAS-tauMycGFP/ fra3,brat^{11}; UAS-bratMyc/egGAL4
Figure 3	brat/+ eg>FraΔC	brat^{11},UAS-tauMycGFP/+; egGal4, UAS-FraΔCHA/+
	brat/+ + UAS-brat eg>FraΔC	brat^{11},UAS-tauMycGFP/+; egGal4, UAS-FraΔCHA/UAS-bratHA
	brat/+ + UAS-brat^{GD} eg>FraΔC	brat^{11},UAS-tauMycGFP/+; egGal4, UAS-FraΔCHA/UAS-bratHA^{GD}
	brat/+ + UAS-bratRD eg>FraΔC	brat^{11},UAS-tauMycGFP/+; egGal4, UAS-FraΔCHA/UAS-bratHARD
Figure 4	brat/+ + UAS-brat eg>FraΔC	brat^{11},UAS-tauMycGFP/+; egGal4, UAS-FraΔCHA/UAS-bratMyc
	brat/+ + UAS-brat^{ΔNHL} eg>FraΔC	brat^{11},UAS-tauMycGFP/+; egGal4, UAS-FraΔCHA/UAS-brat^{ΔNHL}Myc

	brat/+ + UAS-brat$^{\Delta CC}$ eg>Fra∆C	*brat^{11},UAS-tauMycGFP/+; egGal4, UAS-Fra∆CHA/UAS-brat$^{\Delta CC}$Myc*
	brat/+ + UAS-brat$^{\Delta BB}$ eg>Fra∆C	*brat^{11},UAS-tauMycGFP/+; egGal4, UAS-Fra∆CHA/UAS-brat$^{\Delta BB}$Myc*
	brat/+ + UAS-brat$^{\Delta BB1}$ eg>Fra∆C	*brat^{11},UAS-tauMycGFP/+; egGal4, UAS-Fra∆CHA/UAS-brat$^{\Delta BB1}$Myc*
	brat/+ + UAS-brat$^{\Delta BB2}$ eg>Fra∆C	*brat^{11},UAS-tauMycGFP/+; egGal4, UAS-Fra∆CHA/UAS-brat$^{\Delta BB2}$Myc*
Figure 5	*Apc2/+ eg>Fra∆C*	*UAS-A5CD8GFP/+; Apc2^{g10}/egGal4, UAS-Fra∆CHA*
	brat/+; Apc2/+ eg>Fra∆C	*brat^{11},UAS-tauMycGFP/+; egGal4, UAS-Fra∆CHA/Apc2^{g10}*
	brat(-); Apc2(-)	*brat^{11},UAS-tauMycGFP/brat^{11}; Apc2^{g10},egGal4/Apc2^{g10}*
	Df(2L)Exel6168/+ eg>Fra∆C	*Df(2L)Exel6168/UAS-tauMycGFP; egGal4, UAS-Fra∆CHA/+*
	brat/+ eg>Fra∆C	*brat^{11},UAS-tauMycGFP/+; egGal4, UAS-Fra∆CHA/+*
	brat/+; +UAS-Apc2 eg>Fra∆C	*brat^{11},UAS-tauMycGFP/+; egGal4, UAS-Fra∆CHA/UAS-Apc2GFP*
	Apc2(-)	*UAS-tauMycGFP/+; Apc2^{g10},egGal4/Apc2^{g10}*
	arm/+ eg>Fra∆C	*arm^{8}/X; UAS-A5CD8GFP/+; egGal4, UAS-Fra∆CHA/+*
	+UAS-arm eg>Fra∆C	*UAS-A5CD8GFP/+; egGal4, UAS-Fra∆CHA/UAS-armGFP*
	+UAS-armS10 eg>Fra∆C	*UAS-A5CD8GFP/+; egGal4, UAS-Fra∆CHA/UAS-armS10*
	+UAS-∆TCF eg>Fra∆C	*UAS-A5CD8GFP/+; egGal4, UAS-Fra∆CHA/UAS-pan.dTCFDeltaN*
	+UAS-TCF eg>Fra∆C	*UAS-A5CD8GFP/+; egGal4, UAS-Fra∆CHA/UAS-pan.dTCF*
	arm/+; brat/+	*arm^{8}/X; brat^{11}, UAS-tauMycGFP/+; egGal4/+*
	brat/+; eg>arm-GFP	*brat^{11}, UAS-tauMycGFP/UAS-armGFP; egGal4/+*
Figure 6	*+/+; eg>Apc2-GFP,EB1-RFP*	*UAS-EB1-RFP/+; UAS-Apc2-GFP/egGAL4*

	+/+; eg>Apc2-GFP	UAS-Apc2GFP/egGAL4
	brat(-) eg>Apc2-GFP	brat^{11}/brat^{11};UAS-Apc2GFP/egGAL4
Figure S1	brat RNAm, eg>GFP	UAS-tauMycGFP/+; egGAL4/+
Figure S2	WT	elavGAL4/+
	elav>brat	elavGAL4/+; UAS-bratMyc/+
	eg>FraΔC	UAS-A5CD8GFP/+; egGal4, UAS-FraΔCHA/+
	+UAS-brat eg>FraΔC	UAS-A5CD8GFP/+; egGal4, UAS-FraΔCHA/UAS-bratMyc
Figure S3	eg>brat-HA	+/+; UASbratHA/egGAL4
	eg>UAS-brat^{GD}	+/+; UASbratHA^{GD}/egGAL4
	eg>UAS-bratRD	+/+; UASbratHARD/egGAL4
	eg>UAS-bratMyc	+/+; egGAL4/UAS-bratMyc
	eg>UAS-brat$^{\Delta NHL}$	+/+; egGal4/UAS-brat$^{\Delta NHL}$Myc
	eg>UAS-brat$^{\Delta CC}$	+/+; egGal4/UAS-brat$^{\Delta CC}$Myc
	eg>UAS-brat$^{\Delta BB}$	+/+; egGal4/UAS-brat$^{\Delta BB}$Myc
	eg>UAS-brat$^{\Delta BB1}$	+/+; egGal4/UAS-brat$^{\Delta BB1}$Myc
	eg>UAS-brat$^{\Delta BB2}$	+/+; egGal4/UAS-brat$^{\Delta BB2}$Myc
Figure S4	+/+; src-GFP	+/+; scr64b-GFP/+
	brat^{11}(-); scr-GFP	brat^{11}/brat^{11}; scr64b-GFP/+

Figure S5	*+/+; eg>brat-HA*	*UAS-bratHA/egGal4*
	Apc2(-) eg>brat-HA	*$Apc2^{g10}$,UAS-bratHA/$Apc2^{g10}$, egGal4*
